# Consistent host and organ occupancy of phyllosphere bacteria in a community of wild herbaceous plant species

**DOI:** 10.1038/s41396-019-0531-8

**Published:** 2019-10-17

**Authors:** Julien Massoni, Miriam Bortfeld-Miller, Ludwig Jardillier, Guillem Salazar, Shinichi Sunagawa, Julia A. Vorholt

**Affiliations:** 1 Department of Biology, Institute of Microbiology, ETH Zurich, Vladimir-Prelog-Weg 1-5/10, 8093 Zurich, Switzerland; 2 Center for Adaptation to a Changing Environment, ETH Zurich, 8092 Zurich, Switzerland; 3 Unité d’Ecologie, Systématique et Evolution, CNRS UMR 8079, Université Paris-Sud, 91405 Orsay, France

**Keywords:** Microbial ecology, Microbiome

## Abstract

Bacteria colonizing the aerial parts of plants (phyllosphere) are linked to the biology of their host. They impact plant–pathogen interactions and may influence plant reproduction. Past studies have shown differences in composition and structure of the leaf, flower, and host microbiota, but an investigation of the impact of individual taxa on these variations remains to be tested. Such information will help to evaluate disparities and to better understand the biology and evolution of the plant–microbe associations. In the present study, we investigated the community structure, occupancy of host and organ, and the prevalence of phyllosphere bacteria from three host species collected at the same location. Almost all (98%) of bacterial taxa detected in the phyllosphere were not only shared across leaves and flowers, or different plant species but also had a conserved prevalence across sub-environments of the phyllosphere. We also found nonrandom associations of the phylogenetic diversity of phyllosphere bacteria. These results suggest that the phyllosphere microbiota is more conserved than previously acknowledged, and dominated by generalist bacteria adapted to environmental heterogeneity through evolutionary conserved traits.

## Introduction

Angiosperms form the largest clade of extant land plants on earth and colonize a multitude of environments ranging from deserts to aquatic ecosystems. In addition, they are hosts to diverse communities of prokaryotes and eukaryotes, which themselves impact the ecology and evolution of plants [1–3]. Leaf and flower microbiota have triggered recent interest due to the characterization of new functional roles of these microorganisms. For example, floral bacteria impact pollinator behaviors and may influence plant communications [4, 5], leaf bacteria can offer protection against pathogens [6–9], and plant microbiota influence organ development in the phyllosphere [10, 11]. Because these effects might change fitness and gene flow among populations of plants, phyllosphere bacteria may have played an important role in the diversification and adaptation of flowering plants.

Furthermore, because the phyllosphere is itself a heterogeneous environment as a result of different hosts, organs, and variations within each compartment, questions on bacterial adaptation and evolution arise. For example, how did these habitat differences promote host and organ specialist or generalist strategies? [12]. This important aspect in community ecology relates with the niche breadth concept. Thus, characterizing distribution of phyllosphere bacteria across plant species and organs will not only help to identify constraints on their community assembly and ecological specialization, but also to provide a basis for understanding the adaptation of bacteria to this environment, and processes such as speciation and extinction [13–15]. Furthermore, due to the feedback of the microbiota to the plant, such data are crucial for investigating plant bacteria coevolution.

Bacterial distribution across host species and plant organs results from the capacity of taxa to reach (actively or passively), maintain themselves, and multiply in these environments. Previous authors characterized leaf and floral communities in wild plant species and found differences in taxonomical compositions [16–27]. Even if their goal was not to characterize plant-organ occupation of individual bacterial taxa, their results suggest that phyllosphere communities comprise more organ specialists than generalists, as most of the taxa were detected in only flowers or leaves of studied plant species [16, 17, 19]. With regard to host species, previous authors found contrasting results. Epiphytic and endophytic bacterial populations of leaves had most of their OTUs being detected in specific host species [17, 20, 21, 24, 26, 27]. However, when authors focused on the genus *Methylobacterium* they found communities with similar compositions across different host species [25], and similar bacterial communities were found on surfaces of photosynthetic stems of different cacti species [23]. Observed differences across phyllosphere compartments could be partly explained by colonization via insect vectors [28–31], pollen grains [32], or migration via plant tissues and vessels [33, 34]. Such modes of transmission might be constraint and thus limit migration capacities of some members of phyllosphere communities. While these studies provided first insights into the distribution of bacteria across leaves and flowers of different plant species, it is important to investigate this aspect at the level of individual bacterial taxa.

Another important open aspect of the phyllosphere microbiota is the evolutionary history of bacterial taxa that may constrain community assembly in their environment. Microbial communities can gather nonrandom phylogenetic diversity [35], which illustrates both the importance of ecological similarity among taxa that form them, and the presence of evolutionary conservatism of associated traits [36, 37]. Kembel et al. [21] showed that bacterial populations of leaves in tropical trees host more closely related taxa than if phylogenetic relationships did not matter (phylogenetic clustering). They also found a positive correlation between the strength of this pattern and the growth rate of the hosts. For this reason, the assembly of leaf-bacterial communities of fast growing herbaceous species [38] should also be strongly constrained by the evolution of phyllosphere bacteria. Flowers and leaves differ in their carbon source availability, volatile-organic-compound emissions, exposure to environmental variation, and microbial context [17, 19]. These differences, in addition of the high heterogeneity of the floral environment, might lead to different selection pressures on the bacterial diversity and might be illustrated by distinct phylogenetic structures.

In the present work, we investigated the bacterial distribution across plant organs, host species, and the influence of bacterial evolution on leaf and floral communities. Specifically we asked: (i) To what degree do phyllosphere bacteria actively or passively reach both leaves and flowers (i.e., organ occupancy)? (ii) To what degree do phyllosphere bacteria actively or passively reach different hosts (i.e., host occupancy)? (iii) Does the probability of bacterial presence across organs and hosts vary (i.e., prevalence)? (iv) Do leaf and floral OTUs gather in communities independently of their phylogenetic relationships (i.e., phylogenetic conservatism)? To address these questions, we used a design in which we extensively sampled leaves and flowers of wild herbaceous species growing in a meadow and in close proximity to each other to reduce environmental effects on the observed results. We tested the impact of rarefaction depth to test the effect on the capacity of detection of bacterial diversity. We used modeling approaches to control for potential biases caused by dependencies within the data and the limit of detection of OTUs in leaf and floral samples. Results are discussed in light of the different modes of displacement of bacteria and with regard to the current knowledge of evolution of generalism and specialism in heterogeneous environments.

## Results

### Leaf and floral bacterial diversity

We characterized the leaf and floral microbiota of *Ranunculus acris* (Ranunculaceae), *Holcus lanatus* (Poaceae), and *Trifolium pratense* (Fabaceae) that we randomly collected in a 30 × 15 m plot in two time series: seven time points at the end of May (daily over 1 week) and six time points at the end of June (Table S1). For the last two time points in June, we collected two additional species of Poaceae (*Briza media*, *Bromus erectus*), one additional Fabaceae (*Lotus corniculatus*), and three additional Asteraceae (*Centaurea jacea*, *Erigeron annuus*, and *Leucanthemum vulgare*). In total, more than 280 samples were analyzed.

**Fig. 1:**
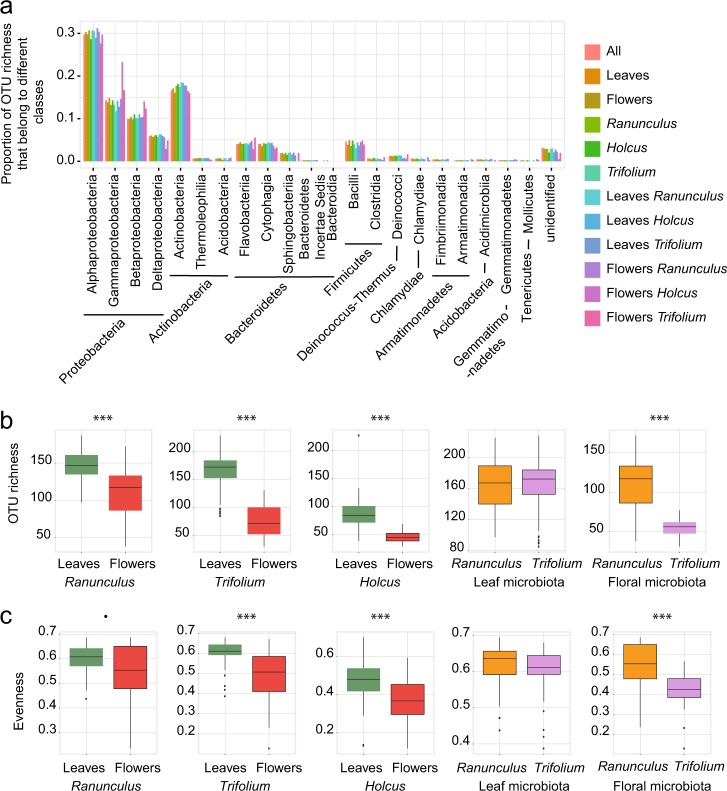
Diversity analyses. **a** Proportions of OTUs belonging to the different bacterial classes in entire dataset and different hosts and organs (samples were rarefied at 6955 reads). **b**, Boxplots of OTU richness in organs and plant species with significance of differences indicated (see details in Table S3). **c** Boxplots of evenness across organs and plant species with significance of differences indicated (see details in Table S3). Significance codes: 0 = ‘***’, 0.001 = ‘**’, 0.01 = ‘*’, 0.05 = ‘.’

Illumina 16S rDNA amplicon sequencing was used to detect taxa. After raw data treatment (see the “Methods” section) our dataset included 7,335,551 reads. The average number of reads per samples was 25,829 in the entire dataset (23,261 and 28,746 reads for leaf and flowers, respectively). *R. acris* and *T. pratense* samples had more reads per sample on average than *H. lanatus* (26,098, 30,642, and 18,001 reads, respectively). We detected 476 OTUs at a 97% similarity threshold (Table S2). These OTUs were members of 21 different classes and 154 genera. The proportions of OTUs among these classes were similar across host species and organs (Fig. 1a). In all host and plant compartments, we noted that the classes Alphaproteobacteria, Gammaproteobacteria, Betaproteobacteria, and Actinobacteria comprised the largest numbers of OTUs. Each of these taxa accounted from 10 to 31% of the OTUs detected in specific plant species, as well as their leaves flowers. In total, 70% of the OTUs detected in our dataset are members of the above-mentioned classes, and 94% when including in addition other classes, Proteobacteria, Bacteroidetes, and Firmicutes. Richness was higher on leaves than on flowers (1.3-fold higher on average for *R. acris*, *p*  value < 0.001; twofold higher for *T. pratense* and *H. lanatus*, *p* values < 0.001; Fig. 1b, Table S3). In *T. pratense* and *H. lanatus*, leaves harbored more even communities compared with flowers (*p* values < 0.001), whereas leaf and flower communities of *R. acris* were more similar regarding evenness (*p* value = 0.03; Fig. 1c, Table S3). When comparing organ communities across host species, we excluded *H. lanatus* because several individual plants had been pooled together in each sample (see the “Methods” section). Leaf communities of *R. acris* and *T. pratense* had similar richness (*p* value = 0.35) and evenness (*p* value = 0.15; Fig. 1b, c, Table S3). Floral communities of *R. acris* were richer (twofold difference on average, *p* value < 0.001) and more even than those of *T. pratense* (*p* value < 0.001; Fig. 1b, c, Table S3).

### Overall structures of bacterial communities of flowers and leaves within and across host species and over time

**Fig. 2:**
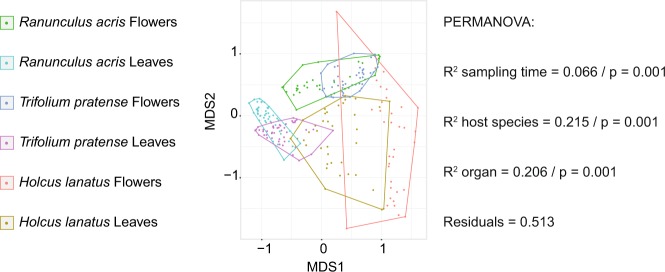
Community structure analyses. Nonmetric multidimensional scaling using Bray–Curtis dissimilarities (stress = 0.15) and results of permutational analysis of variance (PERMANOVA) using “sampling time,” “host species,” and “organs” as explanatory variables. *p,* *p* value

Next, we investigated the overall bacterial community structures (Fig. 2). To do so, we used the Bray–Curtis dissimilarity metric, which accounts for both OTU presence and absence, as well as relative abundances. The permutational multivariate analysis of variance (PERMANOVA) supports a significant difference of communities across time, host species, and organs. Differences in time, host identity, and organ type explained 7, 22, and 21% of the variance, respectively (all *p* values = 0.001, Fig. 2, Supplementary information 1). Further analyses of the impact of time revealed that most of the observed variation could be attributed to the differences between the two time series. However, a significant time effect explained variance in floral and leaf communities of *R. acris* in the first time series (Supplementary information 2).

### Leaf, floral, and host-species occupancy

To estimate the distribution of bacterial taxa across organs and host species, we compared the OTU composition of leaves and flowers of *R. acris*, *T. pratense*, and *H. lanatus*. Furthermore, we took advantage of the large size of our library (25,829 reads on average per sample, see above) to test how rarefaction impacts these comparisons. The presence of OTUs was estimated with a 1000-read incremental rarefaction procedure from 6955 reads (the smallest number per sample) down to 1000 reads. We added an extra dataset to extrapolate the presence of the OTUs detected at 6955 reads from unrarefied samples. It allowed us to take into account the entire capacity of detection for these OTUs as most of samples had more than 6955 reads. In the rest of the text, we refer to these data as the “all-read dataset.”

**Fig. 3:**
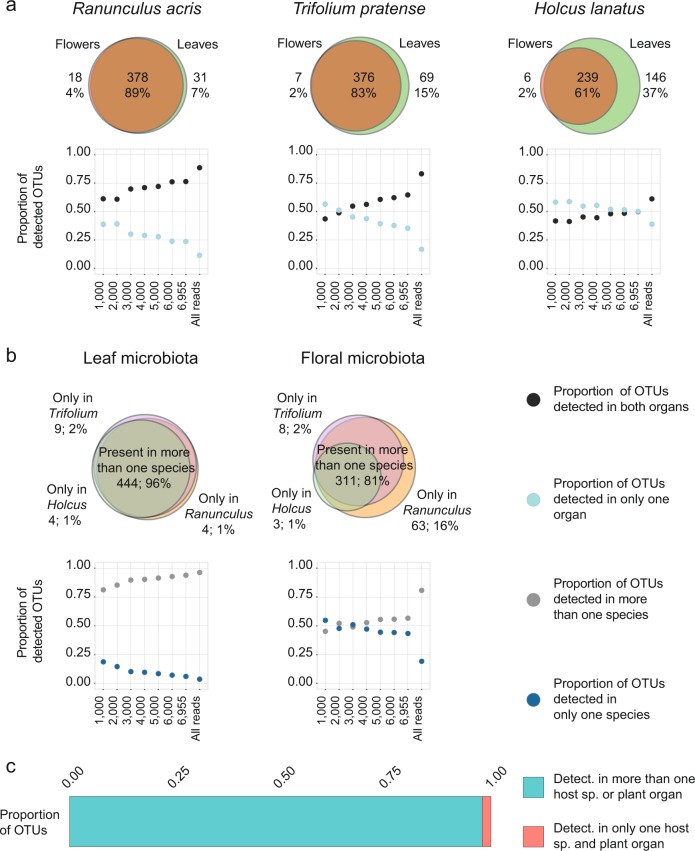
Leaf, floral, and host occupancy analyses. OTU composition overlaps across organs and hosts using absence/presence data. Scatter plots are the evolution of overlaps with the number of reads taken into account per samples. *X* axes of scatter plots are the number of reads per samples used to generate alternative rarefied datasets from 1000 to 6955 reads per samples. “All reads” refers to the dataset in which the presence of the OTUs detected at 6955 reads is extrapolated from unrarefied samples. Venn diagrams were generated from this all-read dataset, and associated values are numbers of OTUs and proportions of tested microbiota that colonize only one organ or host or more than one. **a** Overlaps across flowers and leaves of host-species microbiota. **b** Overlaps across host species of leaf and floral microbiota. **c** Proportion of all OTUs identified in the present study that are detected in more than one host species or plant organ in unrarefied dataset. detect. detected, sp. species

In the all-read dataset, we found that 89, 83, and 61% of the OTUs detected in the microbiota of *R. acris*, *T. pratense*, and *H. lanatus*, respectively, were present in both the leaves and flowers of these species (Fig. 3a). As for organ comparison, in the all-read dataset, 96 and 81% of OTUs were detected in more than one host species in leaf and floral microbiota (Fig. 3b). Among all OTUs identified in the present study, 98% were detected in more than one host or organ (Fig. 3c). The rarefaction procedure highlights the sensitivity of estimations of phyllosphere occupancy to the number of sequences taken into account in individual samples (up to 39% differences) (Fig. 3a, b).

### Prevalence of individual OTUs across leaves, flowers, and plant species

**Fig. 4:**
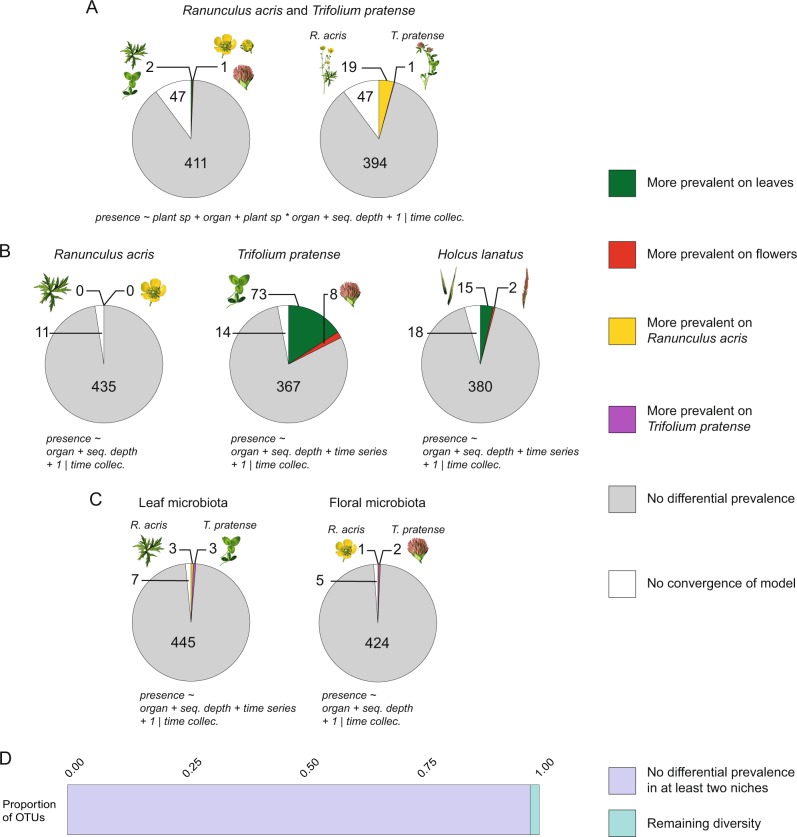
Prevalence analyses across plant organs and hosts. Proportions of OTUs with differential numbers of detection across host species and plant organs were estimated with binomial generalized linear mixed models (Bonferroni corrected *p* values at 0.05 of significance). For each pie chart, the formula of the fitted model used and the number of OTUs in each portion are indicated. **a** Models fitted to the microbiota of *Ranunculus acris* and *Trifolium pratense* analyzed together. **b** Models fitted to host microbiota separately. **c** Models fitted to leaf and floral microbiota separately (*Holcus lanatus* was excluded from this part of analyses; see the “Methods” section). **d** Proportion of OTUs that have comparable prevalence in at least two host/organ combinations; “remaining diversity” refers to OTUs detected in additional plant species collected at the last time point (see the “Methods” section), or OTUs detected in only one host with a differential prevalence across organs.time collec.  time of collection

Subsequently, we investigated whether individual OTUs of the microbiota of *R. acris* and *T. pratense* were more often detected on leaves, flowers, or one of these two host species. *H. lanatus* was excluded because several individual plants were pooled in each sample. We tested for differential prevalence (number of samples in which an OTU is detected) with binomial generalized linear mixed models (GLMM) including “plant species,” “organ,” their interaction, and “sequencing depth” as fixed effects. The time of collection was included as a random effect to correct for potential dependence in the data (see the “Methods” section for details). For 90% of bacteria tested, models converged (Fig. 4a; Table S4). The majority of OTUs did not show significant higher probability of being detected on leaves or flowers (89% at 0.05 Bonferroni corrected significance; Fig. 4a; Table S4). The two OTUs more often detected on leaves belong to the classes Alphaproteobacteria and Cytophagia, and the one more often detected on flowers belongs to the class Bacilli (Table S5). With regard to the prevalence across hosts, most OTUs were also not more often detected on *R. acris* or *T. pratense* (86% at 0.05 Bonferroni corrected significance; Fig. 4a; Table S4). Among the 19 OTUs more often detected in *R. acris*, 12 belonged to Alphaproteobacteria, 3 to Actinobacteria, 2 to Cytophagia, 1 to Betaproteobacteria, and 1 to Thermoleophilia (Table S5). Only one OTU belonging to Alphaproteobacteria was significantly more often detected on *T. pratense* (Table S5).

To better characterize the prevalence of OTUs across leaves and flowers in the microbiota of individual host species, we fitted binomial GLMMs with “plant organ” and “sequencing depth” as fixed effects, and time of collection as a random effect. Because floral samples for *T. pratense* and *H. lanatus* were also collected at the end of June, we included the two time series in the analyses, and “time series” was added as an additional effect (see the “Methods” section for details). For most of the bacteria the models converged (98%, 97%, and 96% in *R. acris*, *T. pratense*, and *H. lanatus*, respectively; Fig. 4b; Table S4). In *R. acris*, no OTUs were significantly more often detected in one organ after Bonferroni correction of the 0.05 significance level. In the microbiota of *T*. pratense and *H. lanatus*, 79 and 92% of OTUs were as prevalent on leaves as on flowers, respectively (Fig. 4b; Table S4). In these two hosts, the number of leaf prevalent OTUs was higher compared with flower prevalent OTUs. Most of the OTUs more often detected on leaves were Alphaproteobacteria, and those more often detected on flowers were Gammaproteobacteria (Table S6).

Finally, to further investigate the host-species prevalence, we also compared the leaf and flower microbiota of *R. acris* and *T. pratense*. Because we pooled several individuals of *H. lanatus* in each sample, we also excluded this species from this part of the study. The formula of the model used was the same as for the microbiota of *T. pratense* and *H. lanatus*, but with the fixed effect “plant organ” replaced by “plant species.” When we compared the leaf communities, the vast majority of OTUs were not more often detected on *R. acris* compared with *T. pratense* (97% of OTUs; Fig. 4c, Table S4). Two Alphaproteobacteria and one Acidobacteria were more often detected on *R. acris*, and two Actinobacteria and one Alphaproteobacteria were more often detected on *T. pratense* (Table S7). In floral communities of these two host species, 98% of OTUs did not show significant differences in their number of detections, 0.2% were more often detected in *R. acris* (one belonging to Alphaproteobacteria), and 0.5% were more often detected in *T. pratense* (two Alphaproteobacteria; Fig. 4c; Tables S4 and S7).

Taking into account all models above, 98% of OTUs present in unrarefied data had the same chance of being detected in more than one organ or host species (Fig. 4d). The remaining 2% were OTUs only detected in one of the three host species mentioned above and more prevalent on leaves or flowers, or only detected in the additional plant species collected at the last time point (see the “Methods” section). The proportion of OTUs with conserved prevalence remained the same when we relaxed the *p* value correction to no correction at all (0.05 significance). In addition, the dominance of the microbiota of *R. acris*, *T. pratense*, *H. lanatus*, leaves, and flowers by OTUs being prevalent across organs and host was also robust to the relaxation of the *p* value correction (Table S4). With regard to their relative abundances, the most abundant OTU of each community was conserved across hosts and organs in all samples. OTUs being more often detected in one environment occupy different positions, but are among the 10 most abundant OTUs in the communities of 42 samples (Figs. S1–7).

### Phylogenetic conservatism

**Fig. 5:**
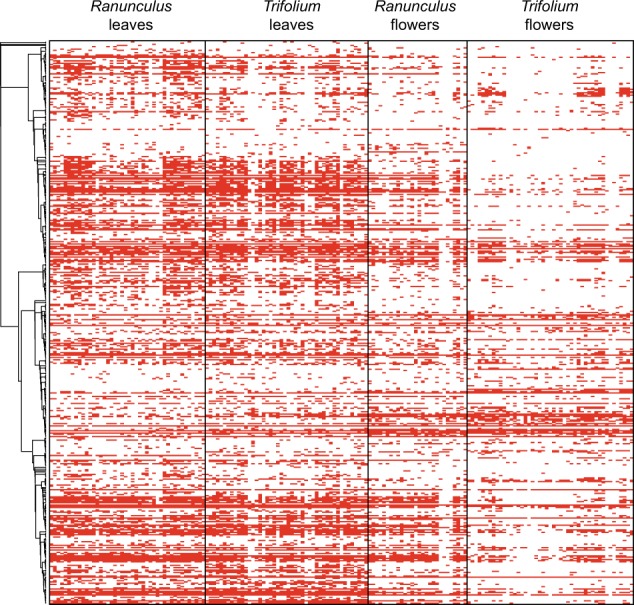
Phylogenetic analyses. Heat map of the detection of OTUs in the dataset rarefied at 6955 reads; positive detections are represented by red squares, and the absence of detections by white squares; rows are clustered according to the best maximum-likelihood tree of all OTUs detected in a non-rarefied dataset

In order to test if the evolutionary history of bacteria detected in *R. acris* and *T. pratense* constrains OTU composition and structure of leaf and floral microbiota, we used null models of phylogenetic community assembly based on Faith’s phylogenetic diversity (PD, [39]). These models assume a community structure that does not involve a combined influence of ecological similarity of species potentially colonizing the environment, and of a phylogenetic conservatism of underlying ecological traits. The method calculates PDs expected under this null assumption. By comparing these expected values with observed PD in real communities of individual samples, the null hypothesis can be rejected at different significance thresholds. Taking into account the phylogeny of all OTUs detected in all plant species sampled in this studies (Fig. S8), all communities of leaves and flowers of *R. acris* and *T. pratense* detected in the 6955 read dataset comprise taxa that are more closely related than under a null model (phylogenetic clustering, *p* value < 0.004), with a mean standardized average effect size of −4.8 (Table S8, Fig. 5).

## Discussion

During the past decade, our knowledge about the diversity of phyllosphere bacteria has significantly increased. Different complementary studies revealed a rich plant microbiota dominated by Proteobacteria [16–24, 26, 27, 40], influencing plant biology [4, 6, 7, 30] and harboring different communities across leaves and flowers [16, 17, 19], and across host species [17, 20, 21, 24, 26, 27] (but see [23, 25]). A more detailed characterization of these variations among phyllosphere communities is crucial to assess if specialization of bacteria to host species and organs underlie these patterns, and if these differences are robust to alternative sampling and analytical approaches. To address these aspects, we estimated distributions of bacteria in leaves and flowers of different plant species. Our sampling approach consisted in collecting three plant species growing in proximity (few meters) and random spatial distributions on a well-defined area. The sampling design allowed us to minimize potential confounding effects of environmental variations. Our results suggest that bacterial communities are less specialized than previously assumed. We found that the vast majority of OTUs detected in this environment do not experience strong limitations in dispersal. This high capacity of dispersal across the phyllosphere is supported by most OTUs being detected across different plant organs and species (Fig. 3). This is in contrast to previous studies that described most bacteria as being present in only leaves or flowers of specific host species [16, 17, 19–21, 24, 26, 27]. Potential reasons for this discrepancy might be the differences in the plant species sampled, the distance between sampled individuals, or the analytical procedures used, including read depth (Fig. 3). Some OTUs of our study might be at the limit of detection; hence the proportion of bacteria only detected in specific hosts or organs in the phyllosphere might still be overestimated.

Most bacterial taxa (98%) were detected with comparable prevalence across leaves and flowers, or among different plant species (Fig. 4). Passive transportation via air flows [41], water [42], aerosols [43], pollen grains [32], solid particles [41], or from soil during plant development [44] could explain the comparable prevalence of these bacteria across plant habitats. In the case of phyllosphere colonization via herbivores or pollinators, specific hosts or organs can be colonized as these vectors only visit some compartments of the phyllosphere [28–31]. However, the equal probabilities of reaching different host species (as indicated by our data) might be explained by insects visiting these different plant species, and by subsequent passive or active migration across organs [42]. Migration via plant vessels or surfaces is another way for bacteria to achieve targeted displacements [33, 34]. In this context, expansion of bacterial populations rather than their entire migration might explain the presence of bacteria in both leaves and flowers.

Although most bacteria were detected in comparable numbers in leaf and flower samples of different host species, we found OTUs that were more prevalent in some compartments than in others (Fig. 4a–c). For these latter “specific” OTUs, we cannot rule out an overestimation of their number due to distinct evenness across host and organ communities of bacteria. In fact, such differences in the distribution of relative abundances can lead to variable detection capacities of specific OTUs. The higher the dominance of some OTUs in communities (decrease of evenness), the higher is the probability to miss less abundant taxa at a given sequencing depth (most abundant OTUs capture more reads). With the first model fitted to the microbiota of *R. acris* and *T. pratense*, we found more OTUs being more prevalent on the first host than on the latter (Fig. 1a). Bacterial communities in *R. acris* are more even than in *T. pratense* leading to a potential higher level of detection of OTU diversity in the first species (Fig. 1c). This pattern of more bacteria being prevalent on *R. acris* is not visible in models fitted to leaf and floral data separately but is present if we relax their *p* value correction (Table S4). Indeed, at 0.05 significance, 23% of floral OTUs are more prevalent on *R. acris* versus 2% being more prevalent on *T. pratense*. In *T. pratense* and *H. lanatus*, we detected some bacteria more often in leaves or flowers, but we did not find any differential prevalence in *R. acris* (Fig. 4b). The floral microbiota of the first two species are less even than their leaf microbiota, whereas all communities of *R. acris* are more similar in this regard (Fig. 1c). Biologically, the higher similarity of leaf and floral bacterial communities in *R. acris* might be explained by the larger exposure of floral communities in the more open flowers of this species. The larger exposure of communities to environmental fluctuations might also explain the variation of diversity through time (Supplementary information 2) and larger OTU richness in this species (Fig. 1b). These unsettled conditions in *R. acris* might prevent the establishment of more dominant taxa due to environmental instability and higher competition. More generally, the presence of less even communities on flowers than on leaves might be explained by the presence of sub-floral micro-environments that could be colonized by a restricted number of bacterial taxa. For example, sugar-rich nectar secretions in *R. acris* and *T. pratense* [45, 46] could lead to a micro-environment where bacteria need to adapt to high osmotic pressures. Bacteria that have the capacity to colonize such floral micro-environments encounter less competition and rich carbon sources potentially allowing them to grow to larger populations than OTUs present in other parts of the flower.

The data generated in this study indicate that bacterial communities in leaves, flowers, and different host species are more similar than previously suggested—at least at one geographic site. This finding coupled with the heterogeneity of these environments with regard to carbon sources [45, 47, 48] and micro-environmental conditions [45, 49] provokes interesting questions regarding the evolution of generalism in the phyllosphere. Generalism in heterogeneous conditions is selected as a function of individual capacities to reach optimal environments (or escape suboptimal ones) and its associated costs [12]. As discussed above, targeted migration or passive transportation can underlie the ubiquity of bacteria through space. If these bacteria have the capacity to select the location they reach, their presence in leaves, flowers, and different host species must be supported by the evolution of a generalist lifestyle. If they cannot actively target leaves, flowers, or host species, theoretical models also support a positive selection for generalism in these populations [12]. Bacteria could have gained the ability to colonize these different hosts and organs either from selection within the phyllosphere (adaptation), other heterogeneous environments (exaptation), or both. Even if some of these mechanisms remain unclear, our measures of phylogenetic clustering in bacterial communities of *R*. acris, *H. lanatus*, and *T. pratense* show that phylogenetic conservatism of functions may drive their modes of assembly (Table S8, Fig. 5b). Because our methods do not guarantee the detection of all rare OTUs in leaf and floral samples, this result applies to their detected fractions. An analysis taking into account any bacteria effectively present in these communities might support a different pattern. In that case, the phylogenetic conservatism measured in the present study would be interpreted as a driver of the assembly of the most abundant fractions of these communities. This conclusion is in line with the results of Kembel et al. [21] regarding leaves of tropical trees. Relevant functions could relate to displacement modes through space, dealing with abiotic conditions, host biology, or biotic interactions (e.g., competition) encountered in this environment. Conservatism suggests that they have low rates of evolution, which might be due to either intrinsic constraints that prevent emergence of alternative strategies, or few functional optima that allow maintenance of high fitness levels in all environments encountered during the life cycles of these bacteria.

The present study suggests that most of the bacteria colonizing the phyllosphere are generalists and have the potential to colonize not only leaves and flowers, but also different plant species. It also suggests that leaf, floral, and different host microbiota might be more conserved than acknowledged before, suggesting a low organ or host-species specialization of phyllosphere microbiota with potential implications for plant-bacteria coevolution and adaptation. Samples taken in the present study were in close proximity and are thus likely to share community members. To evaluate the generalization of the results of the present study, future work applying the same methodology but with replicated blocks across different types of plant communities and at a variety of spatial scales would be of interest. In fact, the different taxonomical compositions of plant microbiota observed in nature might be mainly driven by distance, and site as suggested in other studies [20, 25], and merits further investigations. Finally, we showed that ecological trait similarity, and phylogenetic conservatism of unidentified associated functions could be important drivers of community assembly of bacteria in leaves and flowers.

## Methods

### Collection of samples

The leaves and flowers of individuals of *R. acris*, *T. pratense*, and *H. lanatus* were sampled in a delimited meadow of 30 by 15 m to limit the impact of environmental variation (Fig. S9). The site was located on the Hönggerberg campus of ETH Zurich, Switzerland (47°24′38.27″N: 8°30′25.19″E). These three plant species were homogeneously distributed through the site, and individuals were sampled using randomly generated spatial coordinates. The fact that all random coordinates allowed to sample any plant species during this experiment support an unstructured distribution of all host species through space. To increase the number of samples available for statistical analyses, and to better capture the potentially dynamic composition of communities, we collected two time series at the end of May and June 2016 (Table S1). We sampled every 24 h, with the exception of a few time points that were skipped due to climatic conditions. For the last two time points of sampling, we also collected four individuals of each of the following species: *Briza media*, *Bromus erectus*, *Centaurea jacea*, *Erigeron annuus*, *Leucantherum vulgare*, and *Lotus corniculatus*. For each time point, we collected plant tissues for four individuals of each plant species; leaves and flowers were separately collected into sterile 50 ml Falcon tubes using flamed scissors and forceps. In order to ensure sufficient plant material for subsequent processing, we filled up these tubes with several leaves or flowers of each individual plant of *R. acris* and *T. pratense*. For *H. lanatus*, we pooled organs of several individuals growing at a specific spot, because individuals of this species had only one inflorescence and a few leaves. Samples were flash frozen in liquid nitrogen right after collection and maintained at −80 °C until further processing.

### DNA library preparation and 16S amplicon sequencing

Samples were freeze dried for 48 h and grinded using a TissueLyser II (Quiagen). All samples were normalized to the equivalent to 0.25 ml of dry-tissue powder. Genomic DNA of plant and bacteria was extracted with the FastDNA Spin Kit for Soil (MP Biomedicals, Solon, OH, USA) following the manufacturer’s instructions. After DNA extraction, DNA concentrations were normalized to 5 ng/µl and used for a first PCR with 15 ng of genomic DNA per reaction. Primers 799F and 1193R were used to target the variable regions V5–V7 of the bacterial 16S rDNA, but avoid amplification of homologous sequence in the chloroplast [50, 51]. All reactions were conducted in triplicate with the following PCR conditions and the DFS-Taq DNA polymerase (Bioron, Ludwigshafen, Germany): 2 min at 94 °C, 35 cycles of 30 s at 94 °C, 30 s at 55 °C, 1 min at 72 °C, and a final step of 10 min at 72 °C. After cleaning of PCR products with Antarctic Phosphatase and Exonuclease I *E. coli* (New England BioLabs), a second PCR was conducted to attach barcodes used to multiplex our library with the following conditions: 2 min at 94 °C, nine cycles of 30 s at 94 °C, 30 s at 55 °C, 1 min at 72 °C, and a final step of 10 min at 72 °C. After multiplexing, samples were pooled in equimolar proportions of bacterial DNA. The pooled library was cleaned using AMPure/SeraPure beads to remove unwanted DNA fragments smaller than our amplicon. An electrophoresis gel was run to mechanically separate the 16S rDNA band from the band amplified from the plant mitochondrion which is larger than bacterial 16S rDNA. After DNA purification with the QIAquick Gel Extraction kit (Quiagen), and two additional steps of cleaning for low fragments of DNA, the library was sent for two runs of 2 × 300 paired end sequencing on an Illumina MiSeq system.

In addition to negative controls in the PCRs, blank DNA extractions were processed with the other samples, and two of them were sequenced. After DNA extraction, we added an additional sample of isolated DNA from a well-defined mock community (ZymoBIOMICS Microbial Community Standard; Zymo Research, Irvine, CA, USA). This last control was used to optimize in silico processing of raw sequencing data.

### Demultiplexing and data cleaning

The library was demultiplexed using QIIME [52] with a maximum unacceptable Phred quality score of 20. Another layer of sequencing-error filtering was applied using a maximum value of expected errors of 0.5 using USEARCH v9.2.64 [53]. All singletons were removed, and we tested for a sequencing run effect using the Permutational multivariate analyses of Bray–Curtis variance (PERMANOVA) implemented in the R package Vegan [54]. This test supported an absence of a batch effect, and the two sequencing runs were pooled to increase sequencing depth. Different strategies to filter chimeric sequences were tested, and we evaluated them using the Zymo mock community control. We retained the UPARSE procedure implemented in USEARCH v9.2.64 [55] that led to the composition of the Zymo community being the closest to the theoretical structure provided by the manufacturer. Nonbacterial sequences were removed from data, and an OTU table at 97% similarity was generated. Taxonomical annotations of OTUs were done with the SSU 128 SILVA Parc database as reference [56]. All informatics code used in this process and details about statistics linked with each step are available in Supplementary information  3.

### Noise removal, rarefaction, and outlier exclusion

Before analyses, some additional cleaning steps were implemented. First, all samples with <1000 reads in non-rarefied data were excluded. The two sequenced negative controls had very small amount or reads (19 and 20 reads; Fig. S10; Table S9) with the most abundant OTU having six reads. These results suggest the absence of systematic strong contaminations during the preparation of the library. As the few OTUs found with very small abundance in these controls are present in other samples of the library at much larger abundance, their presence might be due to minor but nonetheless detectable cross-contaminations among samples, or technical biases such as indexing switch happening in multiplexed sequencing. We decided to keep these OTUs in the data not to take the risk of removing biologically relevant information. The number of reads of the first OTU that should not be present in Zymo community (OTU 94: *Carnobacterium*, 63 reads; Fig. S11) was defined as the minimum amount of reads that an OTU should have in the entire non-rarefied dataset to be retained for further analysis. Because this *Carnobacterium* was normally absent from the zymo control, a technical bias should have generated the reads belonging to this OTU. The Zymo control is among the samples with the largest number of reads making this approach conservative (Fig. S10). This procedure equates to removing any OTU with a total abundance across all samples representing <8.5 e^−6^ % of the data. With regard to other OTUs detected with <63 reads in Zymo control, their abundances were low (1–34 reads out of 41,086 reads in sample; Fig. S11), some of them have been excluded from the main dataset by the previous filtering steps, and the remaining ones were kept in the data for the same reasons as mentioned above for the OTUs detected in the two negative controls. After removal of noise, the dataset was rarefied at the depth of the smallest sample (6955 reads; see Fig. S12 for rarefaction curves). Using this dataset, we searched for outliers using OTU richness and Bray–Curtis distances. With the first approach we found different samples that fall below the first quartile −1.5 interquartile range (IQR), or above the third quartile +1.5 IQR, but we did not find any biological argument to exclude them. Using the Bray–Curtis distance, we identified and excluded three samples that strongly differed from others of the same organ and plant species. One leaf sample of *T. pratense* clustered with flowers, and presented a Bray–Curtis distance close to one with all other leaf samples of this plant species. This case might be due to an error that occurred during library preparation. A floral and a leaf sample of *H. lanatus* were also very different from the other samples of this species. The floral sample did not share any OTUs with any other sample of this host species, and these two samples contained OTUs that are known to be present in insects. During the collection of *H. lanatus* in the field, insects were often present on the plant, and some might have been included in collection tubes by accident for these two samples. After removal of these three outliers, this rarefied dataset was used as the reference for further analyses. For the non-rarefied version of the cleaned OTU table, see Table S10. All the code used to generate these additional cleaning steps and to produce the rarefied dataset is available in Supplementary information  4.

### Time of collection, organ type, and plant species as explanatory variables for Bray–Curtis variance among samples

We tested if time, organ type (flowers or leaves), and plant species were significant explanatory variables for differences in OTU contents and OTU relative abundances among samples using PERMANOVA analyses with the Bray–Curtis metric as a measure of sample difference. We also tested for an influence of time in series one, two, and the entire dataset for each host organ separately. All analyses were conducted using the Vegan package in R [54] (Supplementary information 4).

### OTU richness and evenness across organs and plant species

Significant differences of OTU richness and evenness (Pielou’s index [57]) were tested among communities of leaves and flowers, and among communities of host species with data rarefied at 6955 reads. We fitted mixed models, with either “plant organ” or “plant species” as the fixed effect, and “sampling time” as a random slope parameter. Poisson generalized linear mixed models (GLMM) and linear mixed models were used for richness and evenness data, respectively. All analyses were conducted in R [58], and all source code is available in Supplementary information 4. Because organs from several plant individuals of *H. lanatus* were pooled together, we compared OTU richness and evenness among leaf and floral communities of this plant species, but did not compare these values with the ones of *R. acris* and *T. pratense*.

### Leaf, floral, and plant-species occupancy

Comparisons of OTU compositions (absence/presence) of the microbiota of leaves and flowers of *R. acris*, *T. pratense*, and *H*. lanatus were conducted to identify hosts and organs that each bacteria can potentially reach. To test the sensitivity of these results to the rarefaction depth, we used datasets rarefied from 1000 to 6955 reads (Table S11), the latter depth being the smallest sample size of the non-rarefied data. We generated an extra dataset by filtering the non-rarefied OTU table for all OTUs detected at 6955 reads (i.e., excluding those that were not detected at this sequencing depth). We scaled these filtered data into absence–presence to extrapolate the presence of these bacteria from all reads available. We refer to this latter dataset as the “all-read dataset.”

### Prevalence of individual OTUs across leaves, flowers, and plant species

All following analyses were conducted with the unrarefied dataset. We tested if individual OTUs present in *R. acris* or *T. pratense* were more often detected on leaves, flowers, and on one of these two plant species. We modeled their prevalence with binomial GLMMs, with the following fixed effects: “plant species,” “plant organ,” the interaction among “plant species” and “plant organ,” and the centered and rescaled “sequencing depth” of each sample (number of reads). The interaction term among “plant species” and “organ” allows us to take into account a differential influence of the plant organ on the prevalence of OTUs depending on the plant species and *vice versa*. Because our data were collected at different time points, we included the “time of collection” as a random effect in the model (random intercept). A study of the auto-correlation function (ACF) of residuals of fitted models supports the absence of auto-correlation among time points (i.e., time points with specific time lags are not significantly correlated; Supplementary information 4 can be used to regenerate these ACF analyses). Because modeling a random slope in addition of the intercept led to a high rate of failures in convergence (71% of models failed), we decided to not add this level of complexity in models. Because the flowers of *R. acris* were not present in the environment during the collection of the second time series, we restricted our analysis to the first series at the end of May. Using the two series in this model would have introduced an imbalance across the levels of the random effect which can lead to instability of the model [59]. *H. lanatus* was excluded from this analysis, because several plant individuals were pooled in each sample.

To characterize in more detail the prevalence across leaves and flowers, we also fitted binomial GLMMs to the data of each plant-species microbiota separately. Each model comprised “plant organ” and “sequencing depth” as fixed effects in addition of the “time of collection” as a random effect. Because the flowers of *T. pratense* and *H. lanatus* were present during the collection of the two time series, we included “time series” as a fixed effect to take into account the dependence among points at the end of May and at the end of June. We did not add this parameter as a random effect because it did not have the five levels needed to properly estimate the among-population variance [59].

Finally, to better characterize the prevalence across *R. acris* and *T. pratense* in each plant organs, we fitted GLMMs to leaf data (series 1 and 2), and floral data (series 1) separately. The formulas of the models were similar to those used to analyze individual species data, but with the fixed effect “plant organ” replaced by “plant species.”

All the formulas of the models mentioned above are available in Fig. 4. The ratio number of data against number of parameters in models were at least 17 in all models. Before interpreting the significance of each fixed effect in our models, we applied a Bonferroni correction to the 0.05 level of significance. This stringent correction for the multiple testing problem was motivated by the fact that we did not model a random slop which leads to a potential increase of the type I error rate [60], and that *p* values in GLMMs are approximates [61].

### Phylogenetic conservatism in leaf and floral communities of *R. acris* and *T. pratense*

First, we built a phylogeny of all OTUs detected in our environment including those detected in extra plant species collected in the last time points. Because 16S rDNA data provide a limited amount of information to evaluate phylogenetic relationships, the phylogenetic tree of Hug et al. [62] estimated with 16 ribosomal proteins of 3083 genomes was used as a scaffold for our analysis. Several of the OTUs present in our dataset were linked to terminals of their phylogeny in order to impose their relationships to be fixed in accordance with this previous study. To do so, we took the lowest taxonomical level of identification of our OTUs and tried to find these taxa in their phylogenetic tree. The rules of establishment for these correspondences between our data and their tree were applied as followed: if an OTU matched several terminals at a lower taxonomical level, only one was conserved as a constraint; if several OTUs matched one terminal, only one OTU was linked to this tip and the others were freely placed using our 16S rDNA data; when an OTU did not match at the lowest taxonomical level (e.g., genus) but at a higher one (e.g., family), the terminal of the tree was kept as a constraint if no other OTUs in our dataset were constrained at this taxonomical level. We found 113 correspondences between our and their datasets (Fig. S8, Supplementary information 5). As a result, 70 bacterial family positions were constrained to reflect the results of Hug et al. [62]. All OTUs for which we did not find a correspondence with the backbone tree were positioned in the phylogeny using the information contained in the 16S rDNA alignment that we produced with the G-INS-i algorithm of MAFFT v7 [63] (Supplementary information 6). In order to build the final phylogeny, we trimmed the phylogenetic tree of Hug et al. [62] to conserve only terminals that had a correspondence with an OTU in our dataset. Using this tree as a backbone and two extragroups (*Haloferax alexandrinus* and *Guillardia theta* 16S rDNA sequences), we estimated phylogenetic relationships thanks to RAxML v8 [64], a GTR + GAMMA + Inv model of sequence evolution, and 1000 rapid bootstrap inferences followed by a thorough maximum-likelihood search (Fig. S8). Alignment and phylogenetic reconstruction analyses were made on the web server CIPRES [65]. The resulting best likelihood tree is available in Supplementary information 7.

We tested if the evolutionary history of bacteria detected in *R. acris* and *T. pratense* constrains OTU compositions of leaf and floral communities of these plant species using the best maximum-likelihood tree generated in the analysis above. We conducted a null phylogenetic model analysis using the Picante v1.7 package in R [66] to estimate if communities of leaves and flowers of individuals of *R. acris* and *T. pratense* gathered bacterial taxa that were either acquired independently from their phylogenetic relationships or not. In other words, we tested whether members of each bacterial community were more closely related than by chance. Null models assume that functional similarity within the pool of species that potentially colonizes an environment and conservatism of ecological traits in this pool does not affect community assembly. To generate the null model, the general structure of the tree was conserved, taxa names were randomly swapped among tips, and Faiths’ phylogenetic diversity [39] was calculated for each community based on this random phylogeny. We repeated the procedure 1000 times to get null distributions of the PD for each community. By using these null distributions and the observed values of PD in each community based on the real phylogeny, we could calculate a *p* value, and an effect size of the phylogenetic clustering or dispersion for each individual sample.

## Supplementary information

Summary of supplementary information

Supplementary information 1

Supplementary information 2

Supplementary information 3

Supplementary information 4

Supplementary information 5

Supplementary information 6

Supplementary information 7

Supplementary figure 1

Supplementary figure 2

Supplementary figure 3

Supplementary figure 4

Supplementary figure 5

Supplementary figure 6

Supplementary figure 7

Supplementary figure 8

Supplementary figure 9

Supplementary figure 10

Supplementary figure 11

Supplementary figure 12

Supplementary table 1

Supplementary table 2

Supplementary table 3

Supplementary table 4

Supplementary table 5

Supplementary table 6

Supplementary table 7

Supplementary table 8

Supplementary table 9

Supplementary table 10

Supplementary table 11

## Data Availability

Raw sequencing data are available on the European Nucleotide Archive repository: http://www.ebi.ac.uk/ena/data/view/PRJEB34448. All other data generated or analyzed during this study are included in this published article, and its Supplementary information files.

## Source data

Source data 1

## Code availability

All the codes used to demultiplex raw-sequencing data, conduct quality filtering, chimera filtering, OTU definition, and annotation can be found in Supplementary information 3. All the code used for statistical analyses can be found in Supplementary information 4.
