# Supplementary material for: Consistent host and organ occupancy of phyllosphere bacteria in a community of wild herbaceous plant species
Source: ISME J. 2019 Oct 17;14(1):245–58. doi: 10.1038/s41396-019-0531-8 (PMC6908658; doi:10.1038/s41396-019-0531-8)
Supplement: Supplementary file 1 — Summary of supplementary information [file 41396_2019_531_MOESM1_ESM.docx]

SUPPLEMENTARY INFORMATION

**SI 1 (.docx):** Analysis of variance. Detailed results of permutational multivariate analysis of variance with time, host species, and organ as explanatory variables.

**SI 2 (.docx):** Permutational multivariate analyses of variance with sampling time as explanatory variable.

**SI 3 (.html):** Markdown document including bioinformatics code used to demultiplex raw-sequencing data, conduct quality filtering, chimera filtering, OTU definitions, and taxonomical annotations.

**SI 4 (.Rmd; R markdown):** R source code used to conduct final steps of data preparation and statistical analyses. This file is in R Markdown format. It allows to reproduce all analyses on a local computer by following instructions in the “Read me + options” section. Running this script generates an html report of all analyses, in addition of all pdf files, r objects and tables used to make all materials of the present manuscript.

**SI 5 (.txt):** Backbone tree used for phylogenetic analyses.

**SI 6 (.txt):** Alignment of 16S rDNA data used to reconstruct OTU phylogeny.

**SI 7 (.txt):** Best likekihood tree reconstructed with RAxML.

SUPPLEMENTARY TABLES

**Table S1 (.tsv; tab separated format):** Meta data

**Table S2 (.tsv; tab separated format):** Summary of the taxonomy of all OTUs detected in the cleaned dataset

**Table S3 (.xlsx):** Tests for significant differences in OTU richness and community evenness.

**Table S4 (.xlsx):** Proportion of OTUs with and without differential numbers of detection across niches. The level of significance is 0.05 after Bonferroni correction.

**Table S5 (.xlsx):** Summary of the taxonomy of OTUs of microbiotas of *Ranunculus acris* and *Trifolium pratense* with differential numbers of detection across plant organs or hosts. Abbreviations: detect. = detected.

**Table S6 (.xlsx):** Summary of the taxonomy of OTUs with differential numbers of detection across organs of *Trifolium pratense* and *Holcus lanatus*. Abbreviations: More detect. = More often detected; Trif. = *Trifolium pratense*; Hol. = *Holcus lanatus*.

**Table S7 (.xlsx):** Summary of the taxonomy of OTUs with differential numbers of detection across *Ranunculus acris* and *Trifolium pratense* in leaf and floral microbiotas analyzed separately.

**Table S8 (.xlsx):** Output of null phylogenetic models made with the Picante v1.7 R package. *Abbreviations*: ntaxa, number of OTUs in community; pd.rand.mean, mean of Faith’s phylogenetic diversity in null communities; pd.rand.sd, standard deviation of Faith’s phylogenetic diversity in null communities; pd.obs.rank, rank of observed Faith’s phylogenetic diversity vs. null communities; pd.obs.z, standardized effect size of Faith’s phylogenetic diversity vs. null communities; pd.obs.p, p-value of observed PD vs. null communities.

**Table S9 (.xslx):** detected OTUs, associated number of reads, and genus identification in the two sequenced negative controls (unrarefied data).

**Table S10 (.tsv; tab separated format):** Non-rarefied OTU table of the clean dataset.

**Table S11 (.tsv; tab separated format):** OTU tables rarefied from 1,000 to 6,955 reads. All tables are merged. The last two columns allow identification of the rarefaction depth and sample names.

SUPPLEMENTARY FIGURES

**Figures S1-7 (.pdf):** Distributions of OTU relative abundances in each sample colored according to significant differences in numbers of detection in leaves, flowers, or host species. S1-2, Results of the model *presence ~ plant species + organ + plant species * organ + sequencing depth + 1 | time of collection* fitted to microbiotas of *Ranunculus acris* and *Trifolium pratense*; S1, prevalence across organ; S2, prevalence across host species. S3, results of the model *presence ~ organ + sequencing depth + 1 | time of collection* fitted to the microbiota of *Ranunculus acris*. S4-5, results of the model *presence ~ organ + sequencing depth + time series + 1 | time of collection*; S4, fitted to the microbiota of *Trifolium pratense*; S5, fitted to the microbiota of *Holcus lanatus*. S6, results of the model *presence ~ plant species + sequencing depth + time series + 1 | time of collection* fitted to leaf microbiotas of *Ranunculus acris* and *Trifolium pratense*. S7, results of the model *presence ~ plant species + sequencing depth + 1 | time of collection* fitted to floral microbiotas of flowers of *Ranunculus acris* and *Trifolium pratense*. ”Inconclusive” in legend stands for OTUs for which the model did not converge.

**Figure S8 (.pdf):** Best maximum-likelihood tree of all OTUs detected in non-rarefied dataset; tips are colored according to bacterial taxonomy; red branches are phylogenetic relationships constrained by our backbone tree.

**Figure S9 (.pdf):** Picture of the environment sampled in the present study.

**Figure S10 (.pdf):** Distribution of library sizes across samples in the non-rarefied dataset. The red bar is the Zymo control and the two samples in green text are the negative controls used for sequencing.

**Figure S11 (.pdf):** Distribution of abundances in the non-rarefied Zymo control. The red bar is the abundance of the OTU that we considered as not being a real bacterium present in the biological sample, but an OTU generated by technical biases according to the bacterial composition provided by Zymo Research.

**Figure S12 (.pdf):** Rarefaction curves for all samples included in the present study. A, non-rarefied dataset. B, dataset rarefied at 6,955 reads per samples.

SOURCE DATA

Rdata file needed to reproduce analyses with SI 4. It includes the OTU table, OTU sequences, OTU classification, and metadata after demultiplexing, quality filtering, and chimera filtering of raw sequencing data.
