# Supplementary material for: Consistent host and organ occupancy of phyllosphere bacteria in a community of wild herbaceous plant species
Source: ISME J. 2019 Oct 17;14(1):245–58. doi: 10.1038/s41396-019-0531-8 (PMC6908658; doi:10.1038/s41396-019-0531-8)
Supplement: Supplementary file 2 — Supplementary information 1 [file 41396_2019_531_MOESM2_ESM.docx]

**Supplementary information 1: Analysis of variance.**

Results of permutational multivariate analysis of variance with sampling time, host species, and organ as explanatory variables.

Significance codes: 0 ‘***’, 0.001 ‘**’, 0.01 ‘*’, 0.05 ‘.’

Abbreviations: Df “degrees of freedom”; SumsOfSqs “Sums of Squares”; MeanSqs “Mean Squares”; F.Model “F statistics”; R2 “partial R^2^”; Pr(>F) “p-values”.

Permutation: free

Number of permutations: 999

Terms added sequentially (first to last)

|  | Df | SumsOfSqs | MeanSqs | F.Model | R2 | Pr(>F) |
| --- | --- | --- | --- | --- | --- | --- |
| Sampling time | 12 | 4.222 | 0.3518 | 2.379 | 0.06595 | 0.001 *** |
| Host species | 2 | 13.772 | 6.8862 | 46.558 | 0.21513 | 0.001 *** |
| Organ | 1 | 13.188 | 13.1881 | 89.166 | 0.20601 | 0.001 *** |
| Residuals | 222 | 32.835 | 0.1479 |  | 0.5129 |  |
| Total | 237 | 64.017 |  |  | 1 |  |
