# Supplementary material for: Consistent host and organ occupancy of phyllosphere bacteria in a community of wild herbaceous plant species
Source: ISME J. 2019 Oct 17;14(1):245–58. doi: 10.1038/s41396-019-0531-8 (PMC6908658; doi:10.1038/s41396-019-0531-8)
Supplement: Supplementary file 3 — Supplementary information 2 [file 41396_2019_531_MOESM3_ESM.docx]

**Supplementary information 2: Permutational multivariate analyses of variance with time as explanatory variable.**

Significance codes used bellow: 0 ‘***’, 0.001 ‘**’, 0.01 ‘*’, 0.05 ‘.’

Abbreviations : Df “degrees of freedom”; SumsOfSqs “Sums of Squares”; MeanSqs “Mean Squares”; F.Model “F statistics”; R2 “partial R^2^”; Pr(>F) “p-values”.

Floral communities of *Ranunculus acris* for first time series

Permutation: free

Number of permutations: 999

Terms added sequentially (first to last)

|  | Df | SumsOfSqs | MeanSqs | F.Model | R2 | Pr(>F) |
| --- | --- | --- | --- | --- | --- | --- |
| Sampling time | 6 | 1.810 | 0.302 | 2.962 | 0.458 | 0.003 ** |
| Residuals | 21 | 2.139 | 0.102 |  | 0.542 |  |
| Total | 27 | 3.950 |  |  | 1 |  |

Floral communities of *Trifolium pratense* for first time series

Permutation: free

Number of permutations: 999

Terms added sequentially (first to last)

|  | Df | SumsOfSqs | MeanSqs | F.Model | R2 | Pr(>F) |
| --- | --- | --- | --- | --- | --- | --- |
| Sampling time | 6 | 0.426 | 0.071 | 0.809 | 0.195 | 0.748 |
| Residuals | 20 | 1.756 | 0.088 |  | 0.805 |  |
| Total | 26 | 2.183 |  |  | 1 |  |

Floral communities of *Holcus lanatus* for first time series

Permutation: free

Number of permutations: 999

Terms added sequentially (first to last)

|  | Df | SumsOfSqs | MeanSqs | F.Model | R2 | Pr(>F) |
| --- | --- | --- | --- | --- | --- | --- |
| Sampling time | 6 | 1.337 | 0.223 | 0.786 | 0.344 | 0.716 |
| Residuals | 9 | 2.551 | 0.283 |  | 0.656 |  |
| Total | 15 | 3.888 |  |  | 1 |  |

Leaf communities of *Ranunculus acris* for first time series

Permutation: free

Number of permutations: 999

Terms added sequentially (first to last)

|  | Df | SumsOfSqs | MeanSqs | F.Model | R2 | Pr(>F) |
| --- | --- | --- | --- | --- | --- | --- |
| Sampling time | 6 | 0.744 | 0.124 | 1.941 | 0.357 | 0.036 * |
| Residuals | 21 | 1.342 | 0.064 |  | 0.643 |  |
| Total | 27 | 2.086 |  |  | 1 |  |

Leaf communities of *Trifolium pratense* for first time series

Permutation: free

Number of permutations: 999

Terms added sequentially (first to last)

|  | Df | SumsOfSqs | MeanSqs | F.Model | R2 | Pr(>F) |
| --- | --- | --- | --- | --- | --- | --- |
| Sampling time | 6 | 0.728 | 0.121 | 1.190 | 0.263 | 0.278 |
| Residuals | 20 | 2.040 | 0.102 |  | 0.737 |  |
| Total | 26 | 2.769 |  |  | 1 |  |

Leaf communities of *Holcus lanatus* for first time series

Permutation: free

Number of permutations: 999

|  | Df | SumsOfSqs | MeanSqs | F.Model | R2 | Pr(>F) |
| --- | --- | --- | --- | --- | --- | --- |
| Sampling time | 5 | 1.219 | 0.244 | 1.804 | 0.410 | 0.095 . |
| Residuals | 13 | 1.757 | 0.135 |  | 0.590 |  |
| Total | 18 | 2.977 |  |  | 1 |  |
