# Supplementary material for: Consistent host and organ occupancy of phyllosphere bacteria in a community of wild herbaceous plant species
Source: ISME J. 2019 Oct 17;14(1):245–58. doi: 10.1038/s41396-019-0531-8 (PMC6908658; doi:10.1038/s41396-019-0531-8)
Supplement: Supplementary file 4 — Supplementary information 3 [file 41396_2019_531_MOESM4_ESM.html]

SI\_data\_cleaning.md.md


- Sofware used
- Primers (Amplification, indexing, sequencing)
  - Amplification primers
  - Indexing primers
- Controls included in library preparation
- Sequencing output files containing raw data
- Demultiplexing and quality filtering
  - Preparation of the mapping file
  - Validation of the mapping file
  - Testing for the presence of forward indexing primers (plate indexes) in the data set
  - Formating of barcode data in order to make them compatible with the script “split\_libraries\_fastq.py” from QIIME used for demultiplexing
  - Test that all barcodes of the mapping file are all present in data
  - Merging forward and reverse reads
  - Removal of the two bases that belong to the amplification primer 799F
  - Demultiplexing of the library
  - Preparation of clean read files without additional filtering for the two runs separately
  - Preparation of additional clean read files with more quality filtering
  - Merging of read files from the two runs with additional quality filtering
  - Dereplification of datasets
  - Sorting by cluster size and exclusion of singletons
  - Global trimming
- OTU definition and uparse chimera filtering
- Testing of the influence of sequencing runs on data
  - Formatting of sequence names in not-filtered fasta files to keep track of the run that generated the data
  - Merging of the two files final\_reads\_no\_filt.fna
  - Generation of an OTU table from merged runs with tracked sequence names
  - Test for an influence of sequencing runs on data
- Removing of non-bacterial sequences in uparsed OTUs
- Taxonomical annotation of OTUs after uparse filtering
- OTU table after uparse filtering
  - Merging of the two files final\_reads\_no\_filt.fna without tracking of sequencing runs
  - Generation of the table
- Evaluation of the cleaning strategy

Processing of the raw data from sequencing files

# Sofware used

- USEARCH v10 32 bits (does not allows more that 4 Gb of memory)
- QIIME
- USEARCH v9.2 64 bits (When more than 4 Gb of memory was needed):

# Primers (Amplification, indexing, sequencing)

## Amplification primers

799F: AACMGGATTAGATACCCKG

1193R: ACGTCATCCCCACCTTCC

## Indexing primers

### Construct for the forward index:

AATGATACGGCGACCACCGAGATCTACACXXXTGGACTGCGACTGGCGAACMGGATTAGATACCCKG

Orange = adapter that will bind the Illumina MiSeq cellflow

Red = index

Black = tail used to fix indexing primers to PCR primers

Green = 799F primer

indexes:

- B5-F1: TGA
- B5-F2: ACT
- B5-F3: GTC
- B5-F4: CAG

### Construct for the reverse index:

CAAGCAGAAGACGGCATACGAGATXXXXXXXXXXXXCAGCCATTTAGTGTCACGTCATCCCCACCTTCC

Orange = adapter that will bind the cellflow

Red = index

Black = tail used to fix indexing primers to PCR products one

Green = 1193R primer

B5-1: TCCCTTGTCTCC

B5-2: ACGAGACTGATT

B5-3: ACCGGTATGTAC

B5-4: TGCATACACTGG

B5-5: TGGTCAACGATA

B5-6: ATCGCACAGTAA

B5-7: GTCGTGTAGCCT

B5-8: TACAGCGCATAC

B5-9: ATCCTTTGGTTC

B5-10: AGTCGAACGAGG

B5-11: ACCAGTGACTCA

B5-12: CCAATACGCCTG

B5-13: GCAACACCATCC

B5-14: AGTCGTGCACAT

B5-15: AGTTACGAGCTA

B5-16: TTGCGTTAGCAG

B5-17: TACGAGCCCTAA

B5-18: TGTCGCAAATAG

B5-19: ACAATAGACACC

B5-20: TCTCTACCACTC

B5-21: CGATCGAACACT

B5-22: ATTGCAAGCAAC

B5-23: AGCGCTCACATC

B5-24: TCGACCAAACAC

B5-25: TGTGTTACTCCT

B5-26: TGCACAGTCGCT

B5-27: TTCTAGAGTGCG

B5-28: ACACCTGCGATC

B5-29: ATTCCTCTCCAC

B5-30: CATCGACGAGTT

B5-31: CACCACAGAATC

B5-32: GGTCTTAGCACC

B5-33: TATCGCGCGATA

B5-34: CTCTACGAACAG

B5-35: CTCCTCCCTTAC

B5-36: CGTGTTATGTGG

B5-37: ATTAGCAGCGTA

B5-38: CAAGTTTCCGCG

B5-39: CCTTGTTCACCT

B5-40: AACCAGCAGATT

B5-41: CTAGAGCTCCCA

B5-42: CACGCAGTCTAC

B5-43: ACAAACATGGTC

B5-44: TCGAAACATGCA

B5-45: TTCCCACCCATT

B5-46: AGCAGAACATCT

B5-47: GAAACATCCCAC

B5-48: CTGTCAGTGACC

B5-49: CGGATCTAGTGT

B5-50: TTCTCCATCACA

B5-51: ATTTAGGACGAC

B5-52: GGTTTAACACGC

B5-53: AGACAGTAGGAG

B5-54: GCAGATTTCCAG

B5-55: AGATGATCAGTC

B5-56: TATCACCGGCAC

B5-57: CCAGATATAGCA

B5-58: GGTCTCCTACAG

B5-59: ACAGCTCAAACA

B5-60: ATAGCGAACTCA

B5-61: AACCGCATAAGT

B5-62: CTTGAGAAATCG

B5-63: CAGTCGTTAAGA

B5-64: CTTCCAACTCAT

B5-65: AATAGCATGTCG

B5-66: AAGTCACACACA

B5-67: CACACAAAGTCA

B5-68: GTTCCTCCATTA

B5-69: CATCAAGCATAG

B5-70: CAAGCCCTAGTA

B5-71: CCTCTGAGAGCT

B5-72: ACAAGAACCTTG

B5-73: TCATTCCACTCA

B5-74: ACCATCCAACGA

B5-75: ATGCCGGTAATA

B5-76: TCAACCCGTGAA

B5-77: TCTGTAGAGCCA

B5-78: TCGGATCTGTGA

B5-79: ACTACCTCTTCA

B5-80: CTATCCAAGTGG

B5-81: AGCCAGTCATAC

B5-82: GAGTTAGCATCA

B5-83: TAAGACTACTGG

B5-84: GTCTCCTCCCTT

B5-85: CTTAGCTACTCT

B5-86: ATCGAATCGAGT

B5-87: CGCGTCAAACTA

B5-88: GATCAACCCACA

B5-89: AGTGTCGATTCG

B5-90: TCCGAGTCACCA

B5-91: AGTCTGTCTGCG

B5-92: AGACAAGCTTCC

B5-93: GATCTAATCGAG

B5-94: TAAACGCGACTC

B5-95: TCACGAGTCACA

B5-96: TTGAACAAGCCA

### Sequencing primers

F read (B5-R1):

TGGACTGCGACTGGCGAACMGGATTAGATACCC

R read (B5-R2):

CAGCCATTTAGTGTCACGTCATCCCCACCTTCC

Indexing Primer B5-index:

GGAAGGTGGGGATGACGTGACACTAAATGGCTG

Indexing primer B5-Fx:

This primer is present into the sequencing kit used.

# Controls included in library preparation

During the library preparation, We processed negative controls of DNA extractions as separated samples. They stayed negative during the entire processing of the library. We included two of them into the second run of sequencing.

After DNA-extraction, we included the Zymo control which is a sample for which we know the composition in term of bacteria (eight human pathogens that should be not be present in our data):

- *Pseudomonas aeruinosa*
- *Escherichia coli*
- *Salmonella enterica*
- *Lactobacillus fermentum*
- *Enterococcus faecalis*
- *Syaphylococcus aureus*
- *Listeria monocytogenes*
- *Bacillus subtilis*

All samples pooled into the library were associated with PCR controls that were all negative.

# Sequencing output files containing raw data

Each sequencing run produced these series of files:

- Undetermined\_S0\_L001\_I1\_001.fastq: contains 12 bases of B5-index barcode (well index)
- Undetermined\_S0\_L001\_I2\_001.fastq: contains 8 bases including the three bases of B5-Fx barcode (plate index)
- Undetermined\_S0\_L001\_R1\_001.fastq: read 1
- Undetermined\_S0\_L001\_R2\_001.fastq: read 2

# Demultiplexing and quality filtering

## Preparation of the mapping file

Columns in initial file:

- #SampleID
- BarcodeSequence
- LinkerPrimerSequence
- R\_idx\_seq
- R\_idx
- Pos\_R\_idx\_plate
- F\_idx
- F\_idx\_seq

## Validation of the mapping file

In linux:

```
validate_mapping_file.py -m path_to_mapping_file -o path_to_strore_output
```

No problems detected with mapping file

## Testing for the presence of forward indexing primers (plate indexes) in the data set

We applied this step to the two sequencing runs separately.

In linux:

```
data="path_to_fastq_file"
cat ${data} | grep "^@M" -A 1 | grep "^@M" -v | grep "\-\-" -v | awk '{print substr($0,0,3)}' | sort | uniq -c | sort -nr > Data_Findex.txt
```

OK we found the four F indexes in high abundance plus an additional one (TCT) generated by the PhiX control used to increase sequence diversity during sequencing.

## Formating of barcode data in order to make them compatible with the script “split\_libraries\_fastq.py” from QIIME used for demultiplexing

Applied separately to the two sequencing runs.

```
extract_barcodes.py --input_type barcode_paired_end -f path_to_Undetermined_S0_L001_I1_001.fastq -r path_to_Undetermined_S0_L001_I2_001.fastq -o path_for_outputs --bc1_len 12 --bc2_len 3 --rev_comp_bc1
```

Outputs:

- barcodes.fastq that contains concatenated 12-base index + 3-base index.
- reads1.fastq
- reads2.fastq

## Test that all barcodes of the mapping file are all present in data

We extracted the concatenated indexes from the file fastqjoin.join\_barcodes.fastq. We also calculate the abundance for each of them in the data.

In linux:

```
data="path_to_barcodes.fastq"
cat ${data} | grep "^@M" -A 1 | grep "^@M" -v | grep "\-\-" -v | awk '{print substr($0,0,15)}' | sort | uniq -c | sort -nr > Data_barcodes.txt
```

In python:

```
import re

from pandas import read_csv

# Removing of spaces at beginning of lines of the file Data_barcodes.tmp, and save a new file Data_Rindex.csv

with open("path_to_Data_barcodes.txt", "r") as barcodefile:
    with open("path_to_Data_barcodes.csv", "w") as newbarcodefile:
        for line in barcodefile:
            new_line = re.sub("^ *", "", line)
            new_line = re.sub(" ", ",", new_line)
            newbarcodefile.write(new_line)

# Loading of the Data_barcodes.csv file

barcodes = read_csv("path_to_Data_barcodes.csv", header=None, sep=",") # Loading of the data

list_samples = read_csv("path_to_list_samples.txt", header=None, sep=",")[0].tolist() # The list of samples that was included in the current sequencing run. If all samples of the mapping are included ignore this raw.

# Test for the presence of the most abundant concatenated indexes present in the data (i.e. in the file Data_barcodes.tmp)

mapping_file = read_csv("path_to_Mapping_file_demulti.csv_corrected.txt", sep="\t")

mapping_file = mapping_file[mapping_file["#SampleID"].isin(list_samples)] # Use this line only if there are less samples in the present run than in the mapping file

barcodes.columns=["abundance", "barcode"] # Naming columns

list_barcodes = set(list(mapping_file['BarcodeSequence']))

barcodes_present = [] # the list of reverse primers that are present in the data and in dominant abundance

for index, row in barcodes.iterrows(): # Test that all barcodes are present in the dataset
    if row["barcode"] in list_barcodes:
        barcodes_present.append(row["barcode"])
```

All barcodes of the mapping file are present in data.

## Merging forward and reverse reads

Applied to the two sequencing runs separately.

In linux:

```
join_paired_ends.py -f /path_to_Undetermined_S0_L001_R1_001.fastq -r path_to_Undetermined_S0_L001_R2_001.fastq -o path_to_store_outputs -b path_to_barcodes.fastq
```

Outputs:

- fastqjoin.join: assembled / joined reads output
- fastqjoin.un1: unassembled / unjoined reads1 output
- fastqjoin.un2: unassembled / unjoined reads2 output
- fastqjoin.join\_barcodes.fastq: the two indexes concatenated to be used with split\_libraries\_fastq.py in conjunction with fastqjoin.join that contains concatenated reads.

Run 1:

- 8,414,531 reads have been merged out of 11,423,279 reads in raw data

Run 2:

- 5,878,397 reads have been merged out of 10,925,825 reads in raw data

## Removal of the two bases that belong to the amplification primer 799F

Applied to the two sequencing runs separately.

In python:

```
from Bio import SeqIO

with open("path_to_trimmed.fastqjoin.join.fastq", "w") as new_file:
    for record in SeqIO.parse("path_to_fastqjoin.join.fastq", "fastq"):
        subrecord = record[2:]
        new_file.write(subrecord.format("fastq"))
```

## Demultiplexing of the library

Applied separately for each sequencing run.

In linux:

```
split_libraries_fastq.py -i path_to_trimmed.fastqjoin.join.fastq -b path_to_fastqjoin.join_barcodes.fastq --barcode_type 15 --max_barcode_errors 1 -q 19 -o path_to_store_outputs -m path_to_Mapping_file_demulti.csv_corrected.txt --store_demultiplexed_fastq --store_qual_scores
```

- Run 1: 5,077,715 reads were recovered
- Run 2: 3,233,616 reads were recovered

## Preparation of clean read files without additional filtering for the two runs separately

### Formating of sequence names in demultiplexed fasta files

Because the sample information in sequence names of the demultiplexed files are not compatible with some versions of USEARCH, we modified them as follow to solve the problem:

- We added a label “sample=” in names of sequences, that is recognized in all versions of USEARCH.
- We replaced spaces in sequence names with “;”, as it is the format presented in USEARCH 9.2 documentation

We did these modifications with the following python script:

```
import re

with open("path_to_seqs.fna", "r") as read_file:
    with open("path_to_seqs_labeled.fna", "w") as new_file:
        for line in read_file:
            if line.startswith(">"):
                x = re.sub(">", "", re.search("^>([a-z]|[0-9]|[A-Z]|\.)+", line).group(0))
                new_line = re.sub("\n", "", line) + " sample=" + x + "\n"
                new_line = re.sub(" ", ";", new_line)
                new_file.write(new_line)
            else:
                new_file.write(line)
```

We made copies of seqs\_labeled.fna and called them final\_reads\_no\_filt.fna.

## Preparation of additional clean read files with more quality filtering

Applied separately to the two runs of sequencing.

### Filtering based on expected number of errors for reads (E)

We filtered our reads with a stringent value of expected errors not superior to 0.5 (E = 0.5).

In linux:

```
soft_stack/soft/apps/USEARCH/9.2.64-i86linux64/bin/usearch -fastq_filter path_to_seqs.fastq -fastaout path_to_filt_seqs.fasta ‑fastqout path_to_filt_seqs.fastq -fastqout_discarded path_to_filt_seqs_discarded.fastq -fastq_maxee 0.5 -threads 4
```

Results for run 1 filtering:

- 100.0% Filtering, 89.1% passed
- 5,077,715 Reads (5.1M)
- 554,391 Discarded reads with expected errs > 0.50
- 4,523,324 Filtered reads (4.5M, 89.1%)

Results for run 2 filtering:

- 100.0% Filtering, 90.2% passed
- 3,233,616 Reads (3.2M)
- 316,594 Discarded reads with expected errs > 0.50
- 2,917,022 Filtered reads (2.9M, 90.2%)

### Formating of sequence names in filtered fasta file

We followed the same procedure as mentioned above.

In Python:

```
import re

with open("path_to_filt_seqs.fasta", "r") as read_file:
    with open("path_to_filt_seqs_labeled.fna", "w") as new_file:
        for line in read_file:
            if line.startswith(">"):
                x = re.sub(">", "", re.search("^>([a-z]|[0-9]|[A-Z]|\.)+", line).group(0))
                new_line = re.sub("\n", "", line) + " sample=" + x + "\n"
                new_line = re.sub(" ", ";", new_line)
                new_file.write(new_line)
            else:
                new_file.write(line)
```

We created a copy of filt\_seqs\_labeled.fna called final\_reads\_filt.fna

## Merging of read files from the two runs with additional quality filtering

### Formatting of sequence names in filtered fasta files to keep track of the run that generated the data

We did these modifications with the following python script:

```
import re

with open("path_to_filt_seqs.fasta", "r") as read_file:
    with open("path_to_filt_seqs_labeled_runX.fna", "w") as new_file:
        for line in read_file:
            if line.startswith(">"):
                x = re.sub(">", "", re.search("^>([a-z]|[0-9]|[A-Z]|\.)+", line).group(0))
                new_line = re.sub("\n", "", line) + " sample=" + x + ".runX" + "\n"
                new_line = re.sub(" ", ";", new_line)
                new_file.write(new_line)
            else:
                new_file.write(line)
```

### Merging of the two files

In linux:

```
cat filt_seqs_labeled_run1.fna filt_seqs_labeled_run2.fna >> final_reads_filt_merged.fna
```

## Dereplification of datasets

```
soft_stack/soft/apps/USEARCH/9.2.64-i86linux64/bin/usearch -fastx_uniques path_to_final_reads_filt_merged.fna -fastaout path_to_derep_final_reads_filt_merged.fna -sizeout
```

Results run 1:

- 4,523,324 sequences, 1,198,164 uniques, 967,686 singletons

Results run 2:

- 2,917,022 seqs, 851,880 uniques, 697,186 singletons

Result merged runs:

- 7,440,346 seqs, 1,891,741 uniques, 1,540,356 singletons

## Sorting by cluster size and exclusion of singletons

With the option “-minsize 2” only clusters of at least two reads are conserved into the data set (exclusion of singleton).

In linux:

```
usearch -sortbysize path_to_derep_final_reads_filt_merged.fna -fastaout path_to_derep_final_reads_filt_merged_sorted.fasta -minsize 2
```

Results run 1:

- Sorting 230,478 sequences

Results run 2:

- Sorting 154,694 sequences

Results for merged runs:

- Sorting 351,385 sequences

At this step, we copied the file “derep\_final\_reads\_filt\_sorted.fasta”, and named this copy “filt\_reads\_forOTUdef.fasta”.

## Global trimming

In our case we are using 2 X 300 bp paired-end sequencing. For that reason we should not get variable sizes for the reads of the same template as the amplicon is around 400 bp.

# OTU definition and uparse chimera filtering

In linux;

```
usearch -cluster_otus path_to_filt_reads_forOTUdef.fasta -otus path_to_filt_otu_uparse.fasta -uparseout path_to_filt_otu_uparse.txt -relabel OTU
```

Outputs:

- FASTA file for the OTU representative sequences: otu\_uparse.fasta
- A tabbed text documenting how the input sequences were classified: otu\_uparse.txt

Results for run 1:

- 959 OTUs, 5,007 chimeras

Results for run 2:

- 894 OTUs, 2,991 chimeras

Results for merged runs:

- 1,296 OTUs, 6,292 chimeras

# Testing of the influence of sequencing runs on data

The idea is to test if I can merge the two runs.

## Formatting of sequence names in not-filtered fasta files to keep track of the run that generated the data

We did it for the two runs.

In python:

```
import re

with open("path_to_seqs.fna", "r") as read_file:
    with open("/path_to_no_filt_seqs_labeled_runx.fna", "w") as new_file:
        for line in read_file:
            if line.startswith(">"):
                x = re.sub(">", "", re.search("^>([a-z]|[0-9]|[A-Z]|\.)+", line).group(0))
                new_line = re.sub("\n", "", line) + " sample=" + x + ".runX" + "\n"
                new_line = re.sub(" ", ";", new_line)
                new_file.write(new_line)
            else:
                new_file.write(line)
```

## Merging of the two files final\_reads\_no\_filt.fna

In linux:

```
cat path_to_no_filt_seqs_labeled_run1.fna path_to_no_filt_seqs_labeled_run2.fna >> path_to_final_reads_no_filt.fna
```

## Generation of an OTU table from merged runs with tracked sequence names

```
soft_stack/soft/apps/USEARCH/9.2.64-i86linux64/bin/usearch -usearch_global path_to_final_reads_no_filt.fna -db path_to_filt_otu_uparse.fasta -otutabout path_to_otutab_filt_uparse.txt -id 0.97 -strand plus
```

Results:

- 100.0% Searching final\_reads\_no\_filt.fna, 93.9% matched
- 7,799,587 / 8,311,331 mapped to OTUs (93.8%)

## Test for an influence of sequencing runs on data

This part is done in R using Bray Curtis distances among samples.

```
# Test of the effect of runs of sequencing on data

library(vegan)
library(stringr)
library(ggplot2)
library(gplots)

# Loading of abundance data

Abundance_data_tsv <- read.csv(file = "path_to_otutab_filt_uparse.txt", header = TRUE, row.names = 1, sep = "\t", check.names = FALSE, na.strings = "na") # We removed the first line from the file that was a comment informing that the file was generated from a biom file

Abundance_data_tsv <- as.data.frame(t(Abundance_data_tsv)) # The format of data has to be Samples X Species

# Creation of meta-data dataframe containing the run information

sample_tsv <- read.csv(file = "path_to_Mapping_file_demulti.csv_corrected.txt", header = TRUE, row.names = 1, sep = "\t", check.names = FALSE, na.strings = "na") # Mapping file that contains metadata for samples

meta_data <- as.data.frame(matrix(data = NA, ncol = 1, nrow = nrow(Abundance_data_tsv)))

row.names(meta_data) <- row.names(Abundance_data_tsv)

tmp<-strsplit(rownames(meta_data),"\\.")
meta_data[,1]<-sapply(sapply(tmp,rev),"[[",1)

# Filtering of data to the samples that were included in the two sequencing runs

samples_run2 <- read.table("path_to_list_samples_run2.txt")

filt_abun_data <- as.data.frame(Abundance_data_tsv[gsub("\\.run1|\\.run2", "", row.names(Abundance_data_tsv)) %in% samples_run2[,1],]) # filtering of the abundance data

filt_abun_data <- as.data.frame(filt_abun_data[-grep("^NTC.+", rownames(filt_abun_data)),])

# Rarefaction

rarecurve(filt_abun_data,step=500,cex=0.4)

rare_filt_abun_data <- rrarefy(filt_abun_data, 5000)

rare_filt_abun_data<-rare_filt_abun_data[which(rowSums(rare_filt_abun_data) == 5000),]

rowSums(rare_filt_abun_data)

rarecurve(rare_filt_abun_data,step=500,cex=0.4)

# filtering for samples that are represented in both run after rarefaction

list_samples <- gsub("\\.run1|\\.run2", "", rownames(rare_filt_abun_data))

list_samples <- list_samples[duplicated(list_samples)]

abun_data_for_test <- rare_filt_abun_data[which(gsub("\\.run1|\\.run2", "", rownames(rare_filt_abun_data)) %in% list_samples),]

# filtering of metadata

filt_meta_data <- as.data.frame(meta_data[row.names(abun_data_for_test),])

row.names(filt_meta_data) <- row.names(abun_data_for_test)

# Permutational MANOVA

adonis(abun_data_for_test~filt_meta_data[,1])
```

Results of the permutational MANOVA with the sequencing run as explanatory variable:

Call:  
adonis(formula = abun\_data\_for\_test ~ filt\_meta\_data[, 1])

Permutation: free  
Number of permutations: 999

Terms added sequentially (first to last)

filt\_meta\_data[, 1]

- Df = 1
- SumsOfSqs = 0.025
- MeanSqs = 0.025435
- F.Model = 0.092284
- R2 = 0.00035
- Pr(>F) = 1

Residuals

- Df = 266
- MeanSqs = 73.315
- F.Model = 0.275619
- R2 = 0.99965

Total

- Df = 267
- MeanSqs = 73.340
- R2 = 1.00000

# Removing of non-bacterial sequences in uparsed OTUs

We used the Greengenes database to align OTUs with the prealigned CoreSet\_2010 dataset. This database includes only 16S rRNA sequences.

All sequences that do not align to the database with a threshold of identity at 75%, and a minimum length of 150 bp, are excluded.

### Alignment

In linux:

```
align_seqs.py -i path_to_filt_otu_uparse.fasta -t path_to_morg_aln_seqs_T_results_at_0.95.fasta.aligned.imputed -p 0.75 -o path_to_output_repertory
```

Outputs:

- \*\_log.txt: summary of results for each sequence separately
- \*\_failure.fasta: all sequences that did not match the criteria used for alignment
- \*\_aligned.fasta: sequences that were kept into the dataset

115 sequences failed to align

When we blast them to NCBI nucleotide database, most of them did produced any hits. The following ones are those for which we got a match:

- OTU296 = algae
- OTU347 = algae
- OTU499 = algae
- OTU557 = plant
- OTU592 = plant
- OTU655 = plant
- OTU661 = Zebrafish
- OTU712 = plant
- OTU773 = plant
- OTU782 = plant
- OTU805 = plant
- OTU882 = plant
- OTU883 = plant
- OTU923 = plant
- OTU934 = plant
- OTU962 = Ovis canadensis
- OTU1057 = plant
- OTU1098 = plant
- OTU1107 = plant
- OTU1130 = insect
- OTU1144 = plant
- OTU1160 = plant
- OTU1163 = plant
- OTU1197 = algae
- OTU1203 = plant

### Removing the alignment

In linux:

```
sed -i 's/-//g' path_to_filt_otu_uparse_aligned.fasta
```

### Renumbering of OTUs

In linux:

```
cat path_to_filt_otu_uparse_aligned.fasta | awk 'BEGIN {n=1}; />/ {print ">OTU_" n; n++} !/>/ {print}' >> path_to_filt_otu_uparse_final.fasta
```

# Taxonomical annotation of OTUs after uparse filtering

In linux:

```
soft_stack/soft/apps/USEARCH/9.2.64-i86linux64/bin/usearch -usearch_global path_to_filt_otu_uparse_final.fasta -db path_to_SILVA_128_SSUParc_tax_silva.fasta -id 0.9 -maxaccepts 20 -maxrejects 500 -strand both -top_hits_only --output_no_hits -blast6out path_to_tax_filt_otu_uparse_final.txt -threads 4
```

Results:

- 100.0% Searching filt\_otu\_uparse\_final.fasta, 97.1% matched

LCA function to deal with the uncertain taxonomic assignations.

In linux:

```
# LCA
function lca(){ cat $1 | sed -e '$!{N;s/^\(.*\).*\n\1.*$/\1\n\1/;D;}' | awk -F ";" '{$NF=""; OFS=";"; print $0}'; return; }

for i in $(cut -f 1 -d $'\t' path_to_tax_filt_otu_uparse_final.txt | sort | uniq); do id=$(grep -m 1 -P $i'\t' path_to_tax_filt_otu_uparse_final.txt | cut -f 3 -d$'\t'); res=$(grep -P $i'\t' path_to_tax_filt_otu_uparse_final.txt | cut -f 2 -d$'\t' | cut -f 1 -d ' ' --complement | lca); echo -e $i'\t'$res'\t'$id; done > path_to_tax_filt_otu_uparse_final_lca.txt
```

A python script to generate a tsv file that summarizes the taxonomy of OTUs:

```
from pandas import read_csv

import pandas as pd

import re, math

init_file = read_csv("path_to_tax_filt_otu_uparse_final_lca.txt", sep = "\t", header = None) # The file produced by the LCA function

init_file = init_file.fillna(value='Unknown;') # Replacement of the NA values by "Unknown"

index = list(init_file[0]) # List of OTUs

columns = ["Domain", "Phylum", "Class", "Order", "Family", "Genus", "Species"] # List of columns

new_file = pd.DataFrame(data = [re.split(";", row) for row in init_file[1]], index=index, columns=columns) # A dataframe that contains the second column of init_file splitted in different columns

new_file.to_csv("path_to_tax_filt_otu_uparse_proc.txt", sep = "\t", na_rep="", header=True) # Writting of a tsv file
```

# OTU table after uparse filtering

## Merging of the two files final\_reads\_no\_filt.fna without tracking of sequencing runs

In linux:

```
cat path_to_/Run_1/final_reads_no_filt.fna path_to_/Run_2/final_reads_no_filt.fna >> path_to_final_reads_no_filt.fna
```

## Generation of the table

In linux:

```
soft_stack/soft/apps/USEARCH/9.2.64-i86linux64/bin/usearch -usearch_global path_to_final_reads_no_filt.fna -db path_to_filt_otu_uparse_final.fasta -otutabout path_to_otutab_filt_uparse.txt -id 0.97 -strand plus
```

Result:

- 100.0% Searching final\_reads\_no\_filt.fna, 93.8% matched
- 7,795,552 / 8,311,331 mapped to OTUs (93.8%)

# Evaluation of the cleaning strategy

We used the Zyo control for which we know the theoretical content to evaluate the quality of the cleaning process.

Real content of the Zymo control:

- *Pseudomonas aeruinosa*
- *Escherichia coli*
- *Salmonella enterica*
- *Lactobacillus fermentum*
- *Enterococcus faecalis*
- *Staphylococcus aureus*
- *Listeria monocytogenes*
- *Bacillus subtilis*

| OTU ID | Identification | Obtained abundance in Zymo |
| --- | --- | --- |
| OTU55 | 7877 | Bacillus subtilis |
| OTU66 | 7611 | Salmonella enterica |
| OTU76 | 7144 | Staphylococcus aureus |
| OTU59 | 6013 | Lactobacillus fermentum |
| OTU73 | 5692 | Listeria monocytogenes |
| OTU701 | 2812 | Enterococcus sp. |
| OTU87 | 1650 | Pseudomonas aeruinosa |
| OTU37 | 1289 | Escherichia coli |
| OTU557 | 756 | Enterococcus faecium |
| OTU94 | 63 |  |
| OTU173 | 34 |  |
| OTU166 | 31 |  |
| OTU654 | 28 |  |
| OTU803 | 19 |  |
| OTU2 | 15 |  |
| OTU49 | 10 |  |
| OTU282 | 7 |  |
| OTU7 | 4 |  |
| OTU3 | 3 |  |
| OTU1 | 2 |  |
| OTU4 | 2 |  |
| OTU9 | 2 |  |
| OTU15 | 2 |  |
| OTU26 | 2 |  |
| OTU23 | 2 |  |
| OTU176 | 2 |  |
| OTU279 | 2 |  |
| OTU5 | 1 |  |
| OTU11 | 1 |  |
| OTU16 | 1 |  |
| OTU12 | 1 |  |
| OTU13 | 1 |  |
| OTU17 | 1 |  |
| OTU21 | 1 |  |
| OTU25 | 1 |  |
| OTU54 | 1 |  |
| OTU783 | 1 |  |
| OTU291 | 1 |  |
| OTU126 | 1 |  |
