# Supplementary material for: Consistent host and organ occupancy of phyllosphere bacteria in a community of wild herbaceous plant species
Source: ISME J. 2019 Oct 17;14(1):245–58. doi: 10.1038/s41396-019-0531-8 (PMC6908658; doi:10.1038/s41396-019-0531-8)
Supplement: Supplementary file 16 — Supplementary figure 8 [file 41396_2019_531_MOESM16_ESM.pdf]

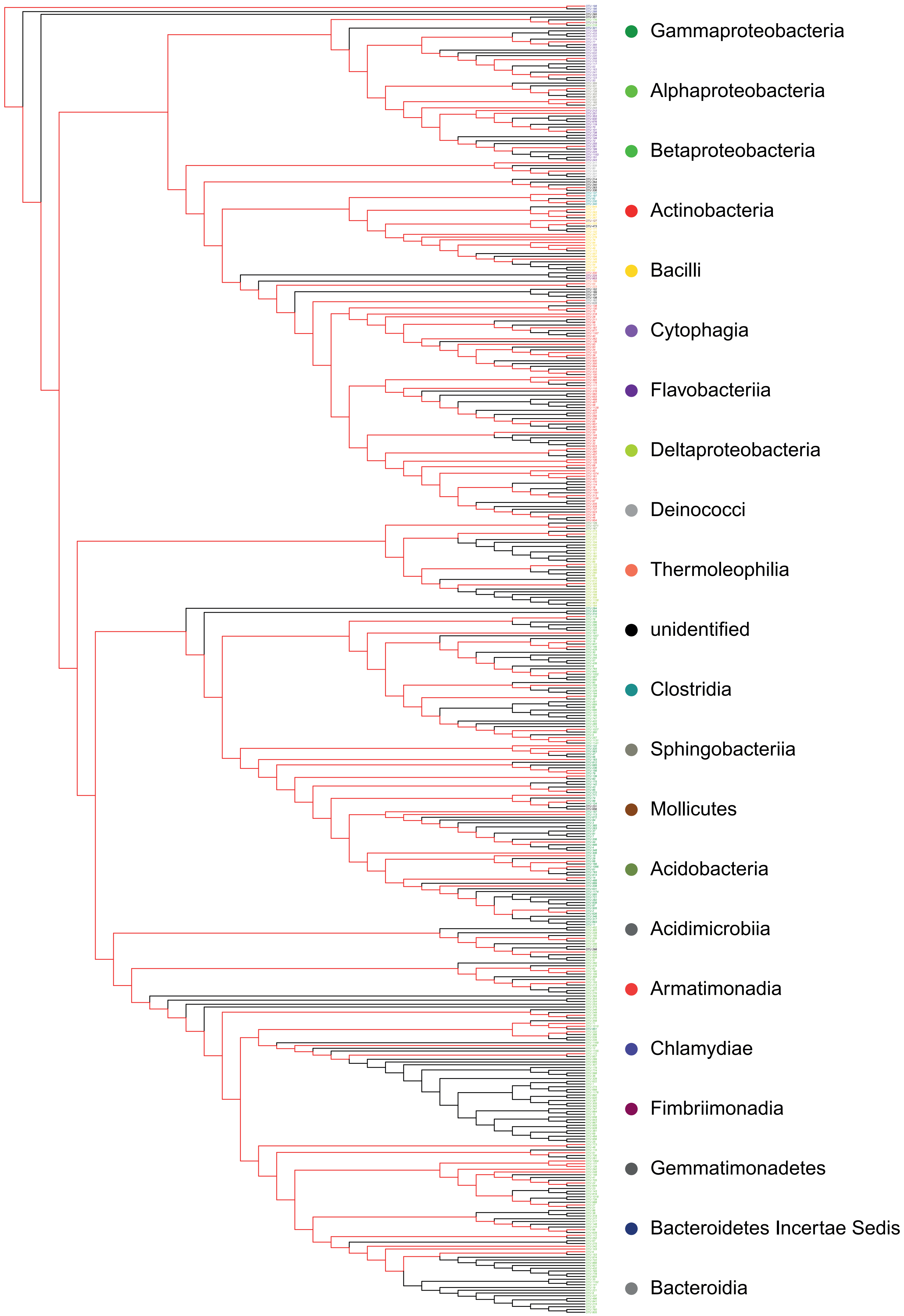
Gammaproteobacteria
Alphaproteobacteria
Betaproteobacteria
Actinobacteria
Bacilli
Cytophagia
Flavobacteriia
Deltaproteobacteria
Deinococci
Thermoleophilia
unidentified
Clostridia
Sphingobacteriia
Mollicutes
Acidobacteria
Acidimicrobiia
Armatimonadia
Chlamydiae
Fimbriimonadia
Gemmatimonadetes
Bacteroidetes Incertae Sedis
Bacteroidia
