# Supplementary figures and images for: Consistent host and organ occupancy of phyllosphere bacteria in a community of wild herbaceous plant species
Source: ISME J. 2019 Oct 17;14(1):245–58. doi: 10.1038/s41396-019-0531-8 (PMC6908658; doi:10.1038/s41396-019-0531-8)

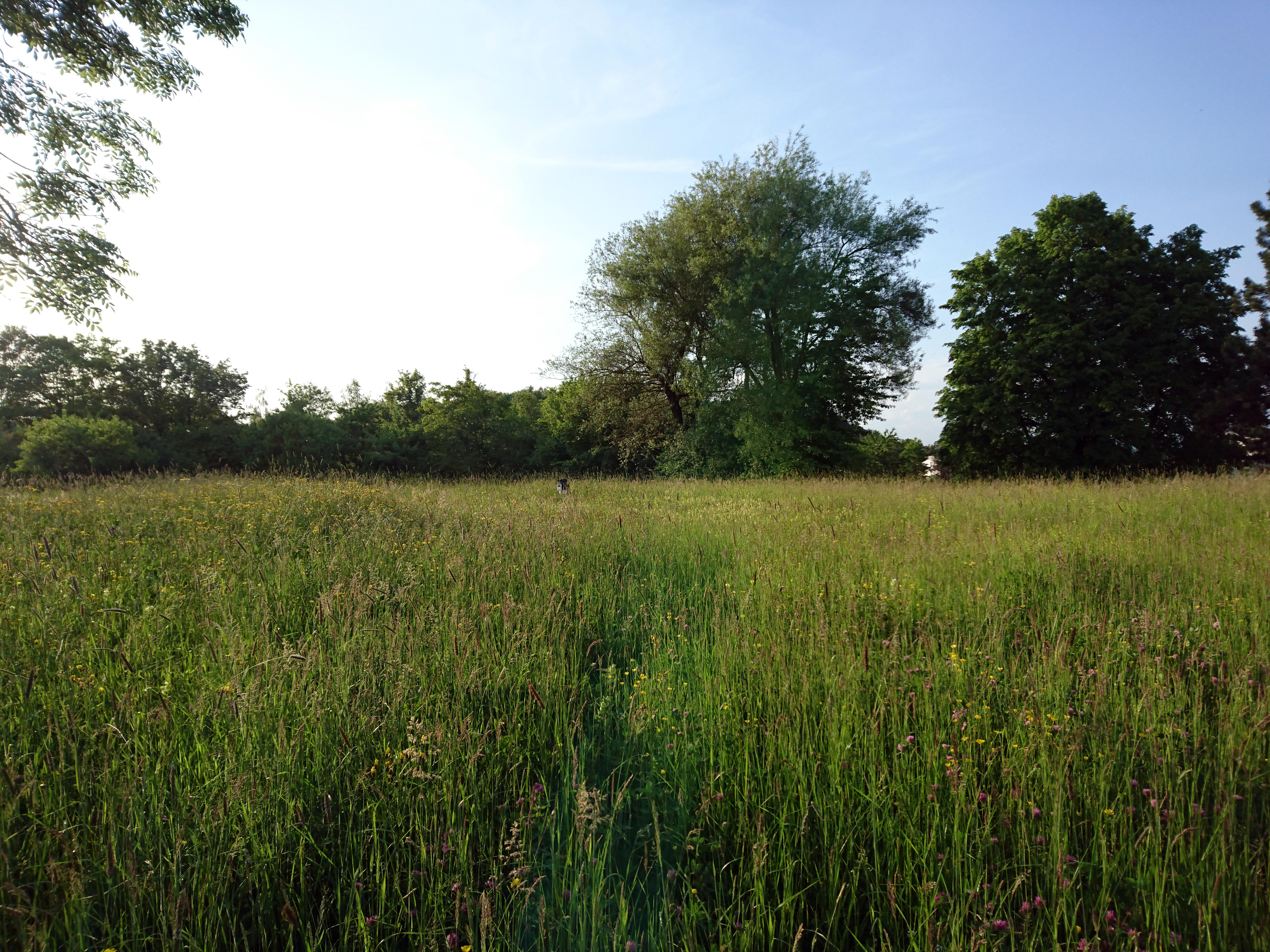

Supplement: Supplementary file 17 — Supplementary figure 9 [file 41396_2019_531_MOESM17_ESM.jpg]

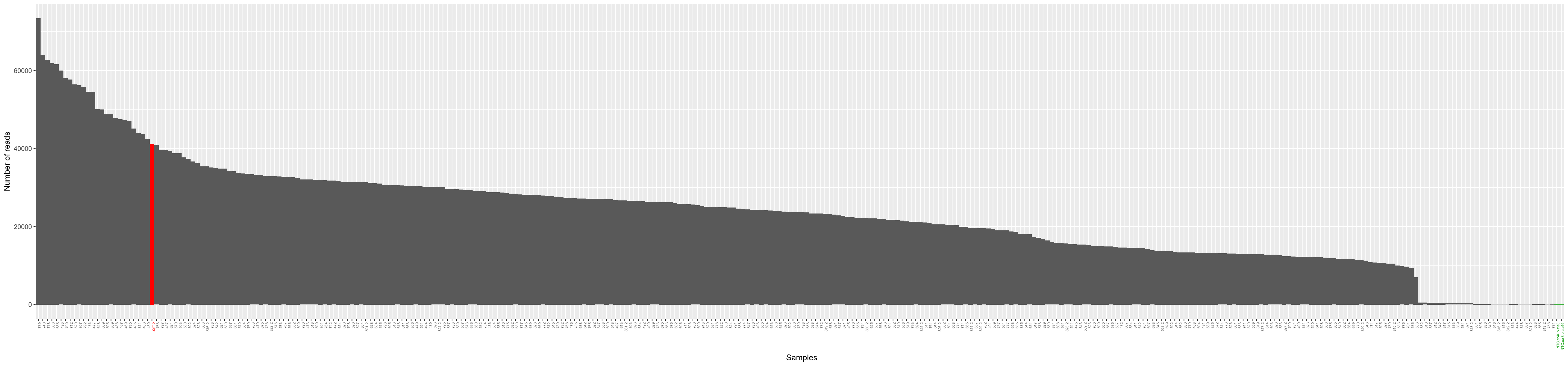

Supplement: Supplementary file 18 — Supplementary figure 10 [file 41396_2019_531_MOESM18_ESM.pdf]

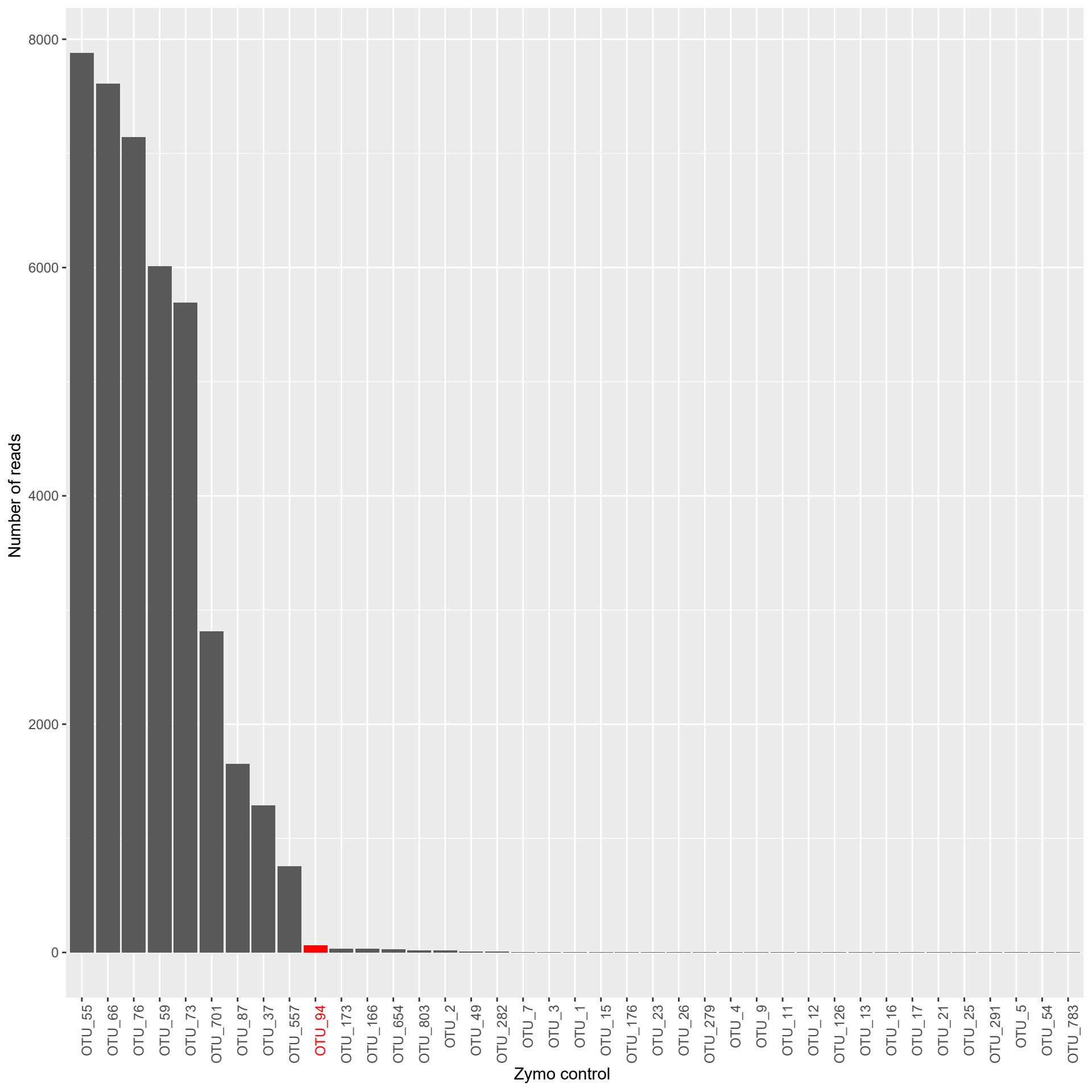

Supplement: Supplementary file 19 — Supplementary figure 11 [file 41396_2019_531_MOESM19_ESM.pdf]

**A**

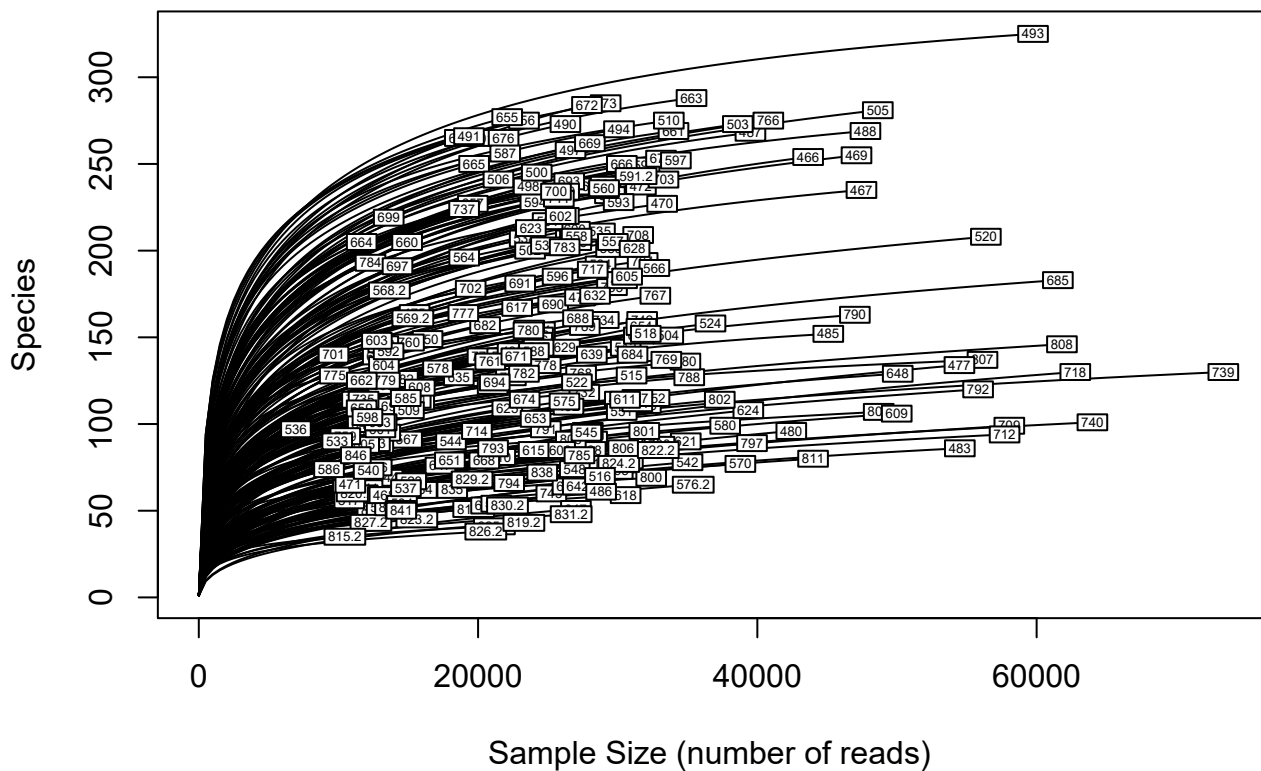

# B

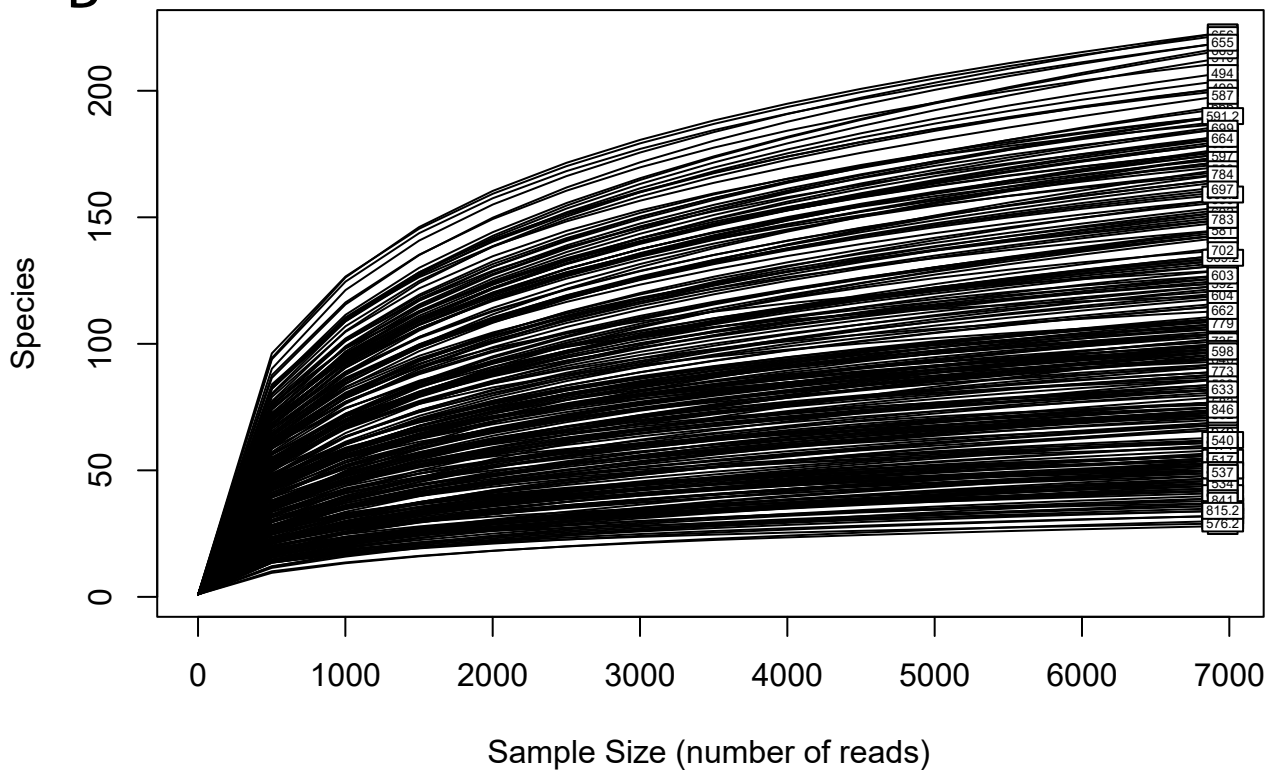

Supplement: Supplementary file 20 — Supplementary figure 12 [file 41396_2019_531_MOESM20_ESM.pdf]
