# Supplementary material for: Consistent host and organ occupancy of phyllosphere bacteria in a community of wild herbaceous plant species
Source: ISME J. 2019 Oct 17;14(1):245–58. doi: 10.1038/s41396-019-0531-8 (PMC6908658; doi:10.1038/s41396-019-0531-8)
Supplement: Supplementary file 10 — Supplementary figure 2 [file 41396_2019_531_MOESM10_ESM.pdf]

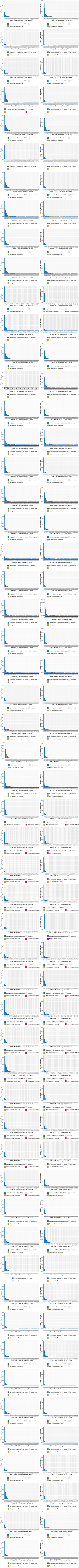
Relative abundance
As prevalent on Ranunculus and Trifolium
Inconclusive
More prevalent on Ranunculus
More prevalent on Trifolium
OTUs of 476 / Ranunculus acris / Flowers
OTUs of 479 / Ranunculus acris / Flowers
OTUs of 482 / Ranunculus acris / Flowers
OTUs of 485 / Ranunculus acris / Flowers
OTUs of 541 / Ranunculus acris / Flowers
OTUs of 544 / Ranunculus acris / Flowers
OTUs of 547 / Ranunculus acris / Flowers
OTUs of 550 / Ranunculus acris / Flowers
OTUs of 569.2 / Ranunculus acris / Flowers
OTUs of 572 / Ranunculus acris / Flowers
OTUs of 575 / Ranunculus acris / Flowers
OTUs of 578 / Ranunculus acris / Flowers
OTUs of 605 / Ranunculus acris / Flowers
OTUs of 608 / Ranunculus acris / Flowers
OTUs of 613 / Ranunculus acris / Flowers
OTUs of 614 / Ranunculus acris / Flowers
OTUs of 617 / Ranunculus acris / Flowers
OTUs of 620 / Ranunculus acris / Flowers
OTUs of 623 / Ranunculus acris / Flowers
OTUs of 628 / Ranunculus acris / Flowers
OTUs of 641 / Ranunculus acris / Flowers
OTUs of 644 / Ranunculus acris / Flowers
OTUs of 647 / Ranunculus acris / Flowers
OTUs of 651 / Ranunculus acris / Flowers
OTUs of 708 / Ranunculus acris / Flowers
OTUs of 711 / Ranunculus acris / Flowers
OTUs of 714 / Ranunculus acris / Flowers
OTUs of 717 / Ranunculus acris / Flowers
OTUs of 466 / Ranunculus acris / Leaves
OTUs of 469 / Ranunculus acris / Leaves
OTUs of 472 / Ranunculus acris / Leaves
OTUs of 475 / Ranunculus acris / Leaves
OTUs of 529 / Ranunculus acris / Leaves
OTUs of 532 / Ranunculus acris / Leaves
OTUs of 535 / Ranunculus acris / Leaves
OTUs of 538 / Ranunculus acris / Leaves
OTUs of 557 / Ranunculus acris / Leaves
OTUs of 560 / Ranunculus acris / Leaves
OTUs of 563 / Ranunculus acris / Leaves
OTUs of 566 / Ranunculus acris / Leaves
OTUs of 581 / Ranunculus acris / Leaves
OTUs of 584 / Ranunculus acris / Leaves
OTUs of 589 / Ranunculus acris / Leaves
OTUs of 590 / Ranunculus acris / Leaves
OTUs of 593 / Ranunculus acris / Leaves
OTUs of 596 / Ranunculus acris / Leaves
OTUs of 599 / Ranunculus acris / Leaves
OTUs of 602 / Ranunculus acris / Leaves
OTUs of 629 / Ranunculus acris / Leaves
OTUs of 632 / Ranunculus acris / Leaves
OTUs of 635 / Ranunculus acris / Leaves
OTUs of 639 / Ranunculus acris / Leaves
OTUs of 693 / Ranunculus acris / Leaves
OTUs of 696 / Ranunculus acris / Leaves
OTUs of 699 / Ranunculus acris / Leaves
OTUs of 702 / Ranunculus acris / Leaves
OTUs of 477 / Trifolium pratense / Flowers
OTUs of 480 / Trifolium pratense / Flowers
OTUs of 483 / Trifolium pratense / Flowers
OTUs of 542 / Trifolium pratense / Flowers
OTUs of 545 / Trifolium pratense / Flowers
OTUs of 548 / Trifolium pratense / Flowers
OTUs of 551 / Trifolium pratense / Flowers
OTUs of 570 / Trifolium pratense / Flowers
OTUs of 573 / Trifolium pratense / Flowers
OTUs of 576.2 / Trifolium pratense / Flowers
OTUs of 580 / Trifolium pratense / Flowers
OTUs of 606 / Trifolium pratense / Flowers
OTUs of 609 / Trifolium pratense / Flowers
OTUs of 611 / Trifolium pratense / Flowers
OTUs of 615 / Trifolium pratense / Flowers
OTUs of 618 / Trifolium pratense / Flowers
OTUs of 621 / Trifolium pratense / Flowers
OTUs of 624 / Trifolium pratense / Flowers
OTUs of 625 / Trifolium pratense / Flowers
OTUs of 642 / Trifolium pratense / Flowers
OTUs of 645 / Trifolium pratense / Flowers
OTUs of 648 / Trifolium pratense / Flowers
OTUs of 652 / Trifolium pratense / Flowers
OTUs of 709 / Trifolium pratense / Flowers
OTUs of 712 / Trifolium pratense / Flowers
OTUs of 715 / Trifolium pratense / Flowers
OTUs of 718 / Trifolium pratense / Flowers
OTUs of 467 / Trifolium pratense / Leaves
OTUs of 470 / Trifolium pratense / Leaves
OTUs of 473 / Trifolium pratense / Leaves
OTUs of 530 / Trifolium pratense / Leaves
OTUs of 533 / Trifolium pratense / Leaves
OTUs of 536 / Trifolium pratense / Leaves
OTUs of 539 / Trifolium pratense / Leaves
OTUs of 558 / Trifolium pratense / Leaves
OTUs of 561 / Trifolium pratense / Leaves
OTUs of 564 / Trifolium pratense / Leaves
OTUs of 568.2 / Trifolium pratense / Leaves
OTUs of 582 / Trifolium pratense / Leaves
OTUs of 585 / Trifolium pratense / Leaves
OTUs of 587 / Trifolium pratense / Leaves
OTUs of 591.2 / Trifolium pratense / Leaves
OTUs of 594 / Trifolium pratense / Leaves
OTUs of 597 / Trifolium pratense / Leaves
OTUs of 600 / Trifolium pratense / Leaves
OTUs of 603 / Trifolium pratense / Leaves
OTUs of 630 / Trifolium pratense / Leaves
OTUs of 633 / Trifolium pratense / Leaves
OTUs of 636 / Trifolium pratense / Leaves
OTUs of 640 / Trifolium pratense / Leaves
OTUs of 694 / Trifolium pratense / Leaves
OTUs of 697 / Trifolium pratense / Leaves
OTUs of 700 / Trifolium pratense / Leaves
OTUs of 703 / Trifolium pratense / Leaves
