# Supplementary material for: Consistent host and organ occupancy of phyllosphere bacteria in a community of wild herbaceous plant species
Source: ISME J. 2019 Oct 17;14(1):245–58. doi: 10.1038/s41396-019-0531-8 (PMC6908658; doi:10.1038/s41396-019-0531-8)
Supplement: Supplementary file 11 — Supplementary figure 3 [file 41396_2019_531_MOESM11_ESM.pdf]

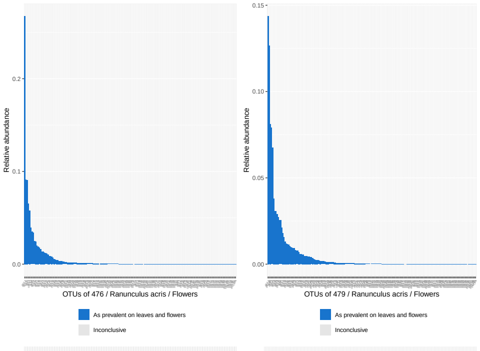
Relative abundance
OTUs of 476 / Ranunculus acris / Flowers
As prevalent on leaves and flowers
Inconclusive
Relative abundance
OTUs of 479 / Ranunculus acris / Flowers
As prevalent on leaves and flowers
Inconclusive

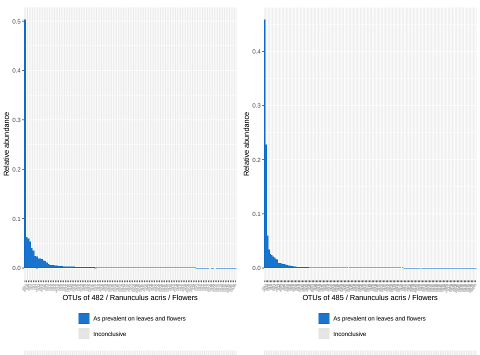
Relative abundance
OTUs of 482 / Ranunculus acris / Flowers
As prevalent on leaves and flowers
Inconclusive
Relative abundance
OTUs of 485 / Ranunculus acris / Flowers
As prevalent on leaves and flowers
Inconclusive

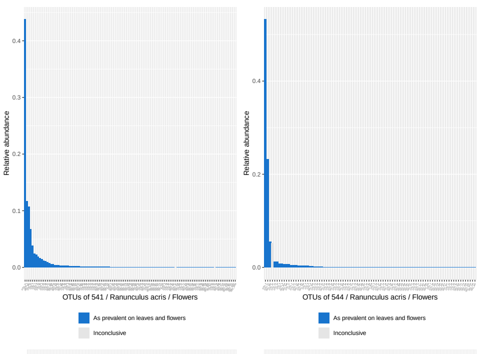
Relative abundance
OTUs of 541 / Ranunculus acris / Flowers
As prevalent on leaves and flowers
Inconclusive
Relative abundance
OTUs of 544 / Ranunculus acris / Flowers
As prevalent on leaves and flowers
Inconclusive

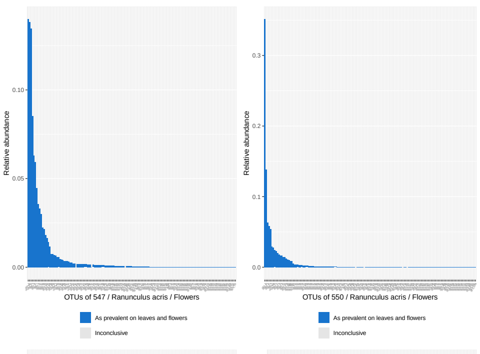
Relative abundance
OTUs of 547 / Ranunculus acris / Flowers
As prevalent on leaves and flowers
Inconclusive
Relative abundance
OTUs of 550 / Ranunculus acris / Flowers
As prevalent on leaves and flowers
Inconclusive

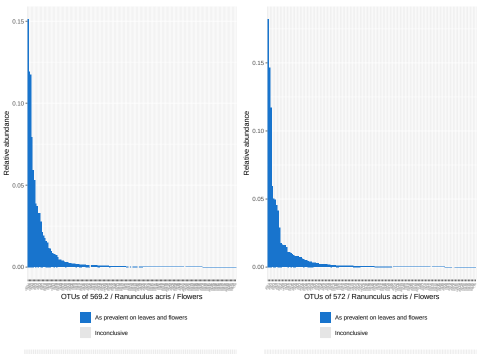
Relative abundance
OTUs of 569.2 / Ranunculus acris / Flowers
As prevalent on leaves and flowers
Inconclusive
Relative abundance
OTUs of 572 / Ranunculus acris / Flowers
As prevalent on leaves and flowers
Inconclusive

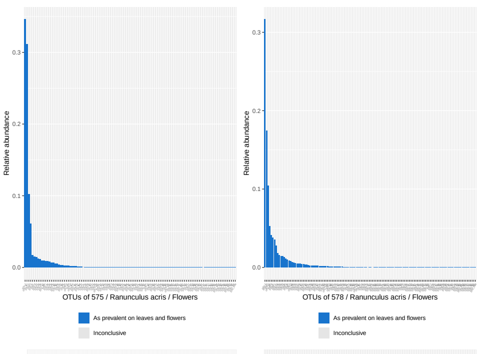
Relative abundance
OTUs of 575 / Ranunculus acris / Flowers
As prevalent on leaves and flowers
Inconclusive
Relative abundance
OTUs of 578 / Ranunculus acris / Flowers
As prevalent on leaves and flowers
Inconclusive

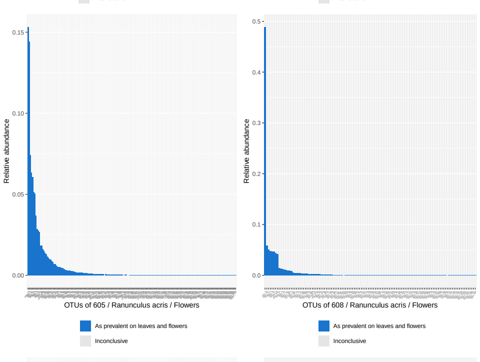
Relative abundance
OTUs of 605 / Ranunculus acris / Flowers
As prevalent on leaves and flowers
Inconclusive
Relative abundance
OTUs of 608 / Ranunculus acris / Flowers
As prevalent on leaves and flowers
Inconclusive

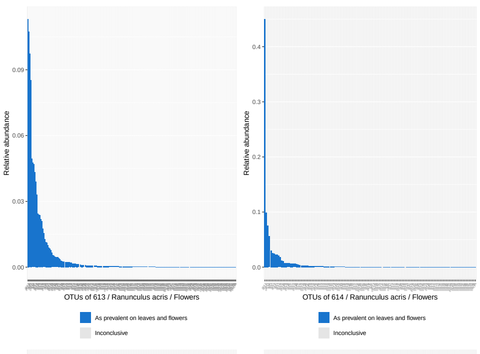
Relative abundance
OTUs of 613 / Ranunculus acris / Flowers
As prevalent on leaves and flowers
Inconclusive
Relative abundance
OTUs of 614 / Ranunculus acris / Flowers
As prevalent on leaves and flowers
Inconclusive

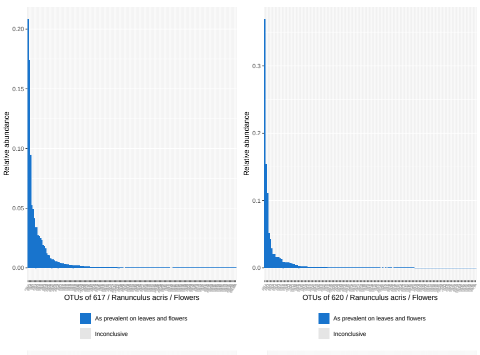
Relative abundance
OTUs of 617 / Ranunculus acris / Flowers
As prevalent on leaves and flowers
Inconclusive
Relative abundance
OTUs of 620 / Ranunculus acris / Flowers
As prevalent on leaves and flowers
Inconclusive

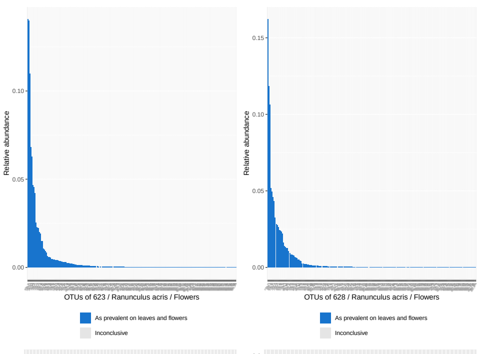
Relative abundance
OTUs of 623 / Ranunculus acris / Flowers
As prevalent on leaves and flowers
Inconclusive
Relative abundance
OTUs of 628 / Ranunculus acris / Flowers
As prevalent on leaves and flowers
Inconclusive

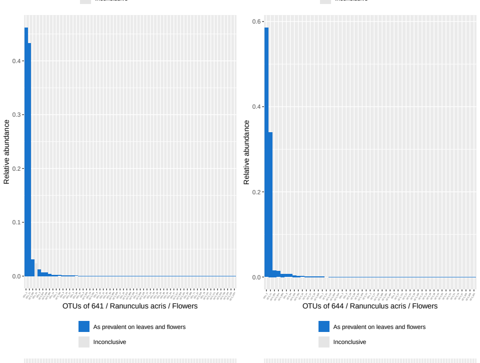
Relative abundance
OTUs of 641 / Ranunculus acris / Flowers
As prevalent on leaves and flowers
Inconclusive
Relative abundance
OTUs of 644 / Ranunculus acris / Flowers
As prevalent on leaves and flowers
Inconclusive

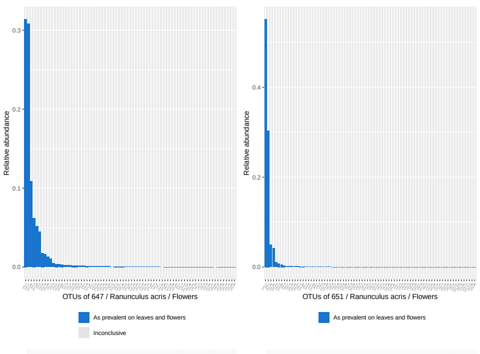
Relative abundance
OTUs of 647 / Ranunculus acris / Flowers
As prevalent on leaves and flowers
Inconclusive
Relative abundance
OTUs of 651 / Ranunculus acris / Flowers
As prevalent on leaves and flowers

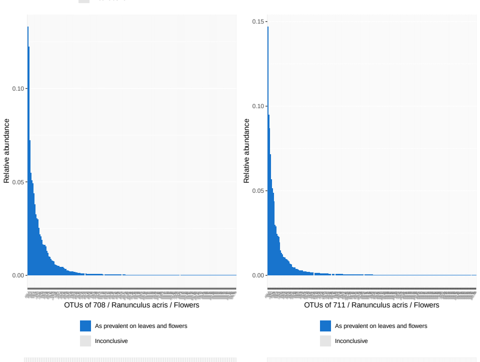
Relative abundance
OTUs of 708 / Ranunculus acris / Flowers
As prevalent on leaves and flowers
Inconclusive
Relative abundance
OTUs of 711 / Ranunculus acris / Flowers
As prevalent on leaves and flowers
Inconclusive

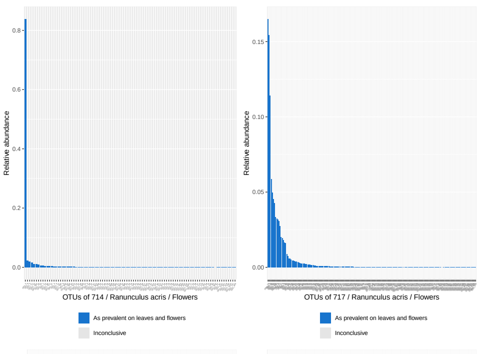
Relative abundance
OTUs of 714 / Ranunculus acris / Flowers
As prevalent on leaves and flowers
Inconclusive
Relative abundance
OTUs of 717 / Ranunculus acris / Flowers
As prevalent on leaves and flowers
Inconclusive

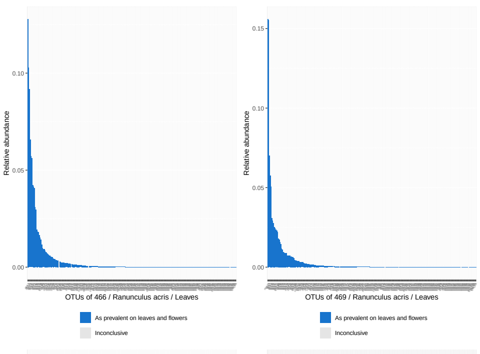
Relative abundance
OTUs of 466 / Ranunculus acris / Leaves
As prevalent on leaves and flowers
Inconclusive
Relative abundance
OTUs of 469 / Ranunculus acris / Leaves
As prevalent on leaves and flowers
Inconclusive

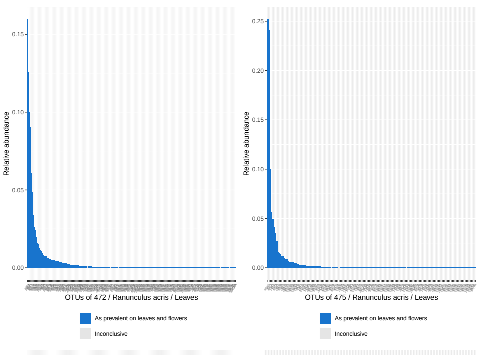
Relative abundance
OTUs of 472 / Ranunculus acris / Leaves
As prevalent on leaves and flowers
Inconclusive
Relative abundance
OTUs of 475 / Ranunculus acris / Leaves
As prevalent on leaves and flowers
Inconclusive

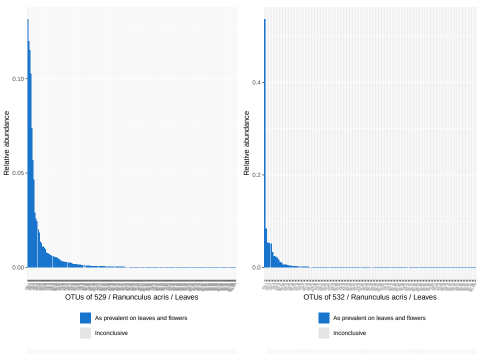
Relative abundance
OTUs of 529 / Ranunculus acris / Leaves
As prevalent on leaves and flowers
Inconclusive
Relative abundance
OTUs of 532 / Ranunculus acris / Leaves
As prevalent on leaves and flowers
Inconclusive

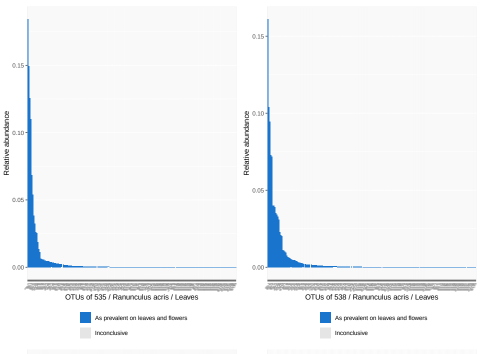
Relative abundance
OTUs of 535 / Ranunculus acris / Leaves
As prevalent on leaves and flowers
Inconclusive
Relative abundance
OTUs of 538 / Ranunculus acris / Leaves
As prevalent on leaves and flowers
Inconclusive

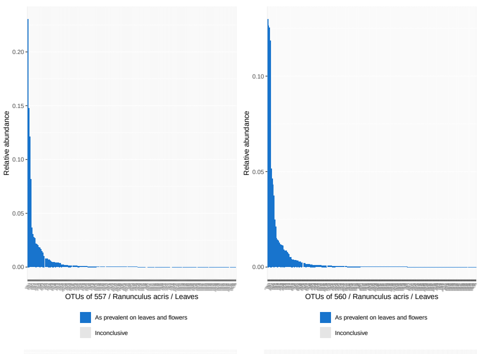
Relative abundance
OTUs of 557 / Ranunculus acris / Leaves
As prevalent on leaves and flowers
Inconclusive
Relative abundance
OTUs of 560 / Ranunculus acris / Leaves
As prevalent on leaves and flowers
Inconclusive

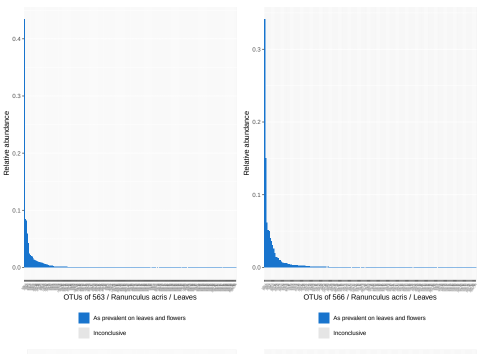
Relative abundance
OTUs of 563 / Ranunculus acris / Leaves
As prevalent on leaves and flowers
Inconclusive
Relative abundance
OTUs of 566 / Ranunculus acris / Leaves
As prevalent on leaves and flowers
Inconclusive

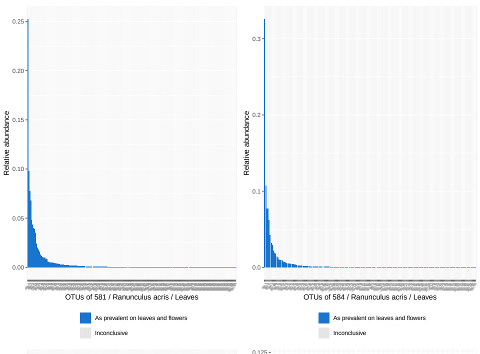
Relative abundance
OTUs of 581 / Ranunculus acris / Leaves
As prevalent on leaves and flowers
Inconclusive
Relative abundance
OTUs of 584 / Ranunculus acris / Leaves
As prevalent on leaves and flowers
Inconclusive

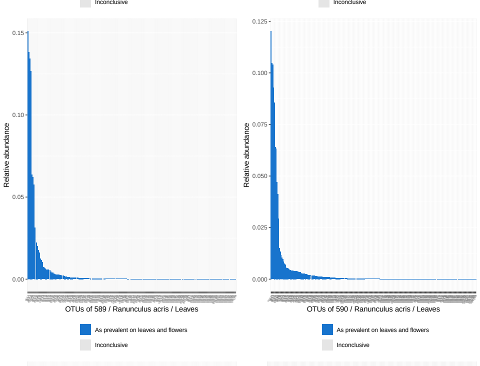
Relative abundance
OTUs of 589 / Ranunculus acris / Leaves
As prevalent on leaves and flowers
Inconclusive
Relative abundance
OTUs of 590 / Ranunculus acris / Leaves
As prevalent on leaves and flowers
Inconclusive

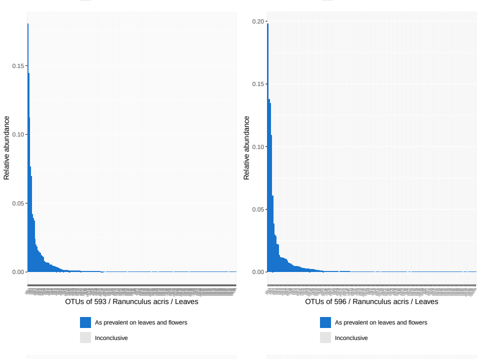
Relative abundance
OTUs of 593 / Ranunculus acris / Leaves
As prevalent on leaves and flowers
Inconclusive
Relative abundance
OTUs of 596 / Ranunculus acris / Leaves
As prevalent on leaves and flowers
Inconclusive

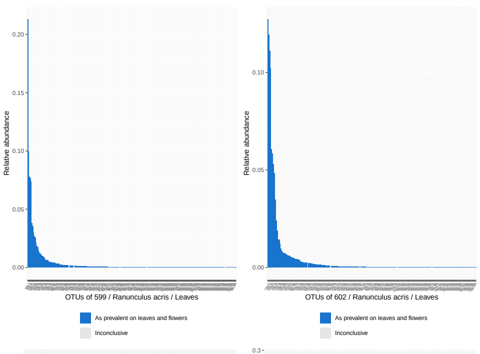
Relative abundance
OTUs of 599 / Ranunculus acris / Leaves
As prevalent on leaves and flowers
Inconclusive
Relative abundance
OTUs of 602 / Ranunculus acris / Leaves
As prevalent on leaves and flowers
Inconclusive

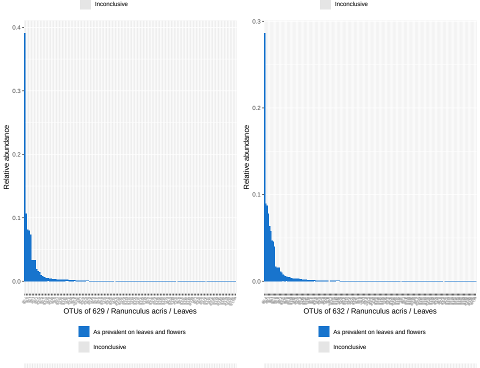
Relative abundance
OTUs of 629 / Ranunculus acris / Leaves
As prevalent on leaves and flowers
Inconclusive
Relative abundance
OTUs of 632 / Ranunculus acris / Leaves
As prevalent on leaves and flowers
Inconclusive

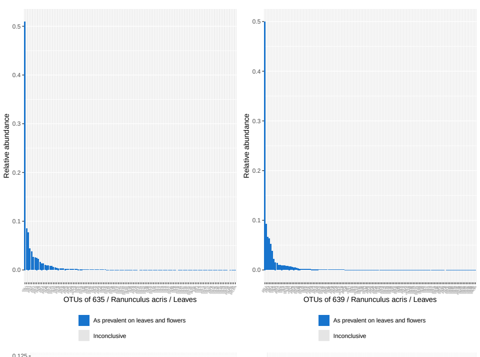
Relative abundance
OTUs of 635 / Ranunculus acris / Leaves
As prevalent on leaves and flowers
Inconclusive
Relative abundance
OTUs of 639 / Ranunculus acris / Leaves
As prevalent on leaves and flowers
Inconclusive

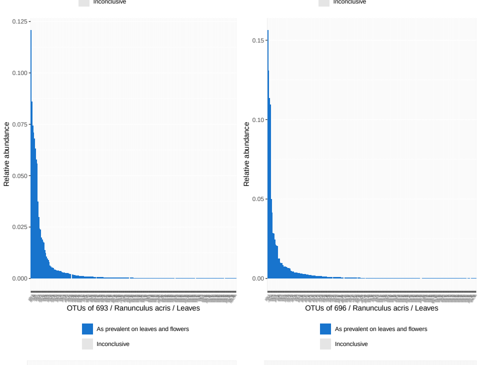
Relative abundance
OTUs of 693 / Ranunculus acris / Leaves
As prevalent on leaves and flowers
Inconclusive
Relative abundance
OTUs of 696 / Ranunculus acris / Leaves
As prevalent on leaves and flowers
Inconclusive

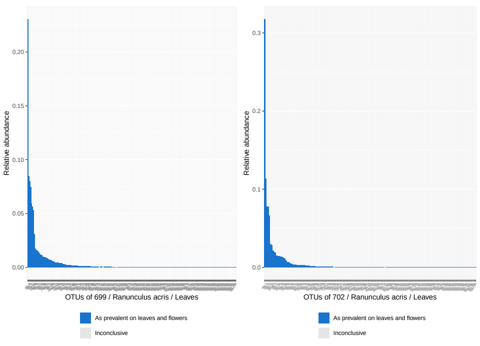
Relative abundance
OTUs of 699 / Ranunculus acris / Leaves
As prevalent on leaves and flowers
Inconclusive
Relative abundance
OTUs of 702 / Ranunculus acris / Leaves
As prevalent on leaves and flowers
Inconclusive
