# Supplementary material for: Consistent host and organ occupancy of phyllosphere bacteria in a community of wild herbaceous plant species
Source: ISME J. 2019 Oct 17;14(1):245–58. doi: 10.1038/s41396-019-0531-8 (PMC6908658; doi:10.1038/s41396-019-0531-8)
Supplement: Supplementary file 12 — Supplementary figure 4 [file 41396_2019_531_MOESM12_ESM.pdf]

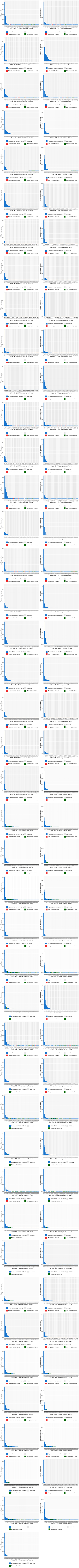

Relative abundance
As prevalent on leaves and flowers
Inconclusive
More prevalent on flowers
More prevalent on leaves
OTUs of 477 / Trifolium pratense / Flowers
OTUs of 480 / Trifolium pratense / Flowers
OTUs of 483 / Trifolium pratense / Flowers
OTUs of 511 / Trifolium pratense / Flowers
OTUs of 513 / Trifolium pratense / Flowers
OTUs of 515 / Trifolium pratense / Flowers
OTUs of 518 / Trifolium pratense / Flowers
OTUs of 520 / Trifolium pratense / Flowers
OTUs of 522 / Trifolium pratense / Flowers
OTUs of 524 / Trifolium pratense / Flowers
OTUs of 527 / Trifolium pratense / Flowers
OTUs of 542 / Trifolium pratense / Flowers
OTUs of 545 / Trifolium pratense / Flowers
OTUs of 548 / Trifolium pratense / Flowers
OTUs of 551 / Trifolium pratense / Flowers
OTUs of 570 / Trifolium pratense / Flowers
OTUs of 573 / Trifolium pratense / Flowers
OTUs of 576.2 / Trifolium pratense / Flowers
OTUs of 580 / Trifolium pratense / Flowers
OTUs of 606 / Trifolium pratense / Flowers
OTUs of 609 / Trifolium pratense / Flowers
OTUs of 611 / Trifolium pratense / Flowers
OTUs of 615 / Trifolium pratense / Flowers
OTUs of 618 / Trifolium pratense / Flowers
OTUs of 621 / Trifolium pratense / Flowers
OTUs of 624 / Trifolium pratense / Flowers
OTUs of 625 / Trifolium pratense / Flowers
OTUs of 642 / Trifolium pratense / Flowers
OTUs of 645 / Trifolium pratense / Flowers
OTUs of 648 / Trifolium pratense / Flowers
OTUs of 652 / Trifolium pratense / Flowers
OTUs of 678 / Trifolium pratense / Flowers
OTUs of 680 / Trifolium pratense / Flowers
OTUs of 682 / Trifolium pratense / Flowers
OTUs of 684 / Trifolium pratense / Flowers
OTUs of 685 / Trifolium pratense / Flowers
OTUs of 688 / Trifolium pratense / Flowers
OTUs of 690 / Trifolium pratense / Flowers
OTUs of 691 / Trifolium pratense / Flowers
OTUs of 709 / Trifolium pratense / Flowers
OTUs of 712 / Trifolium pratense / Flowers
OTUs of 715 / Trifolium pratense / Flowers
OTUs of 718 / Trifolium pratense / Flowers
OTUs of 467 / Trifolium pratense / Leaves
OTUs of 470 / Trifolium pratense / Leaves
OTUs of 473 / Trifolium pratense / Leaves
OTUs of 487 / Trifolium pratense / Leaves
OTUs of 490 / Trifolium pratense / Leaves
OTUs of 493 / Trifolium pratense / Leaves
OTUs of 498 / Trifolium pratense / Leaves
OTUs of 500 / Trifolium pratense / Leaves
OTUs of 502 / Trifolium pratense / Leaves
OTUs of 506 / Trifolium pratense / Leaves
OTUs of 510 / Trifolium pratense / Leaves
OTUs of 530 / Trifolium pratense / Leaves
OTUs of 533 / Trifolium pratense / Leaves
OTUs of 536 / Trifolium pratense / Leaves
OTUs of 539 / Trifolium pratense / Leaves
OTUs of 558 / Trifolium pratense / Leaves
OTUs of 561 / Trifolium pratense / Leaves
OTUs of 564 / Trifolium pratense / Leaves
OTUs of 568.2 / Trifolium pratense / Leaves
OTUs of 582 / Trifolium pratense / Leaves
OTUs of 585 / Trifolium pratense / Leaves
OTUs of 587 / Trifolium pratense / Leaves
OTUs of 591.2 / Trifolium pratense / Leaves
OTUs of 594 / Trifolium pratense / Leaves
OTUs of 597 / Trifolium pratense / Leaves
OTUs of 600 / Trifolium pratense / Leaves
OTUs of 603 / Trifolium pratense / Leaves
OTUs of 630 / Trifolium pratense / Leaves
OTUs of 633 / Trifolium pratense / Leaves
OTUs of 636 / Trifolium pratense / Leaves
OTUs of 640 / Trifolium pratense / Leaves
OTUs of 658 / Trifolium pratense / Leaves
OTUs of 660 / Trifolium pratense / Leaves
OTUs of 664 / Trifolium pratense / Leaves
OTUs of 666 / Trifolium pratense / Leaves
OTUs of 670 / Trifolium pratense / Leaves
OTUs of 672 / Trifolium pratense / Leaves
OTUs of 673 / Trifolium pratense / Leaves
OTUs of 694 / Trifolium pratense / Leaves
OTUs of 697 / Trifolium pratense / Leaves
OTUs of 700 / Trifolium pratense / Leaves
OTUs of 703 / Trifolium pratense / Leaves
