# Supplementary material for: Consistent host and organ occupancy of phyllosphere bacteria in a community of wild herbaceous plant species
Source: ISME J. 2019 Oct 17;14(1):245–58. doi: 10.1038/s41396-019-0531-8 (PMC6908658; doi:10.1038/s41396-019-0531-8)
Supplement: Supplementary file 13 — Supplementary figure 5 [file 41396_2019_531_MOESM13_ESM.pdf]

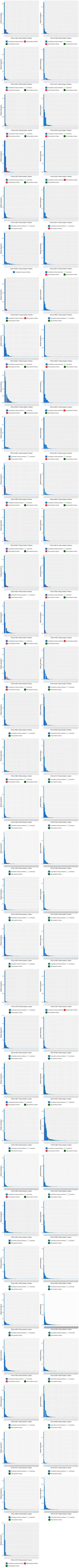
OTUs of 481 / Holcus lanatus / Flowers
OTUs of 486 / Holcus lanatus / Flowers
OTUs of 516 / Holcus lanatus / Flowers
OTUs of 517 / Holcus lanatus / Flowers
OTUs of 519 / Holcus lanatus / Flowers
OTUs of 523 / Holcus lanatus / Flowers
OTUs of 526 / Holcus lanatus / Flowers
OTUs of 612 / Holcus lanatus / Flowers
OTUs of 626 / Holcus lanatus / Flowers
OTUs of 814.2 / Holcus lanatus / Flowers
OTUs of 815.2 / Holcus lanatus / Flowers
OTUs of 817.2 / Holcus lanatus / Flowers
OTUs of 819.2 / Holcus lanatus / Flowers
OTUs of 820.2 / Holcus lanatus / Flowers
OTUs of 822.2 / Holcus lanatus / Flowers
OTUs of 823.2 / Holcus lanatus / Flowers
OTUs of 824.2 / Holcus lanatus / Flowers
OTUs of 825.2 / Holcus lanatus / Flowers
OTUs of 826.2 / Holcus lanatus / Flowers
OTUs of 827.2 / Holcus lanatus / Flowers
OTUs of 828 / Holcus lanatus / Flowers
OTUs of 829.2 / Holcus lanatus / Flowers
OTUs of 830.2 / Holcus lanatus / Flowers
OTUs of 831.2 / Holcus lanatus / Flowers
OTUs of 834 / Holcus lanatus / Flowers
OTUs of 835 / Holcus lanatus / Flowers
OTUs of 838 / Holcus lanatus / Flowers
OTUs of 841 / Holcus lanatus / Flowers
OTUs of 843 / Holcus lanatus / Flowers
OTUs of 844 / Holcus lanatus / Flowers
OTUs of 845 / Holcus lanatus / Flowers
OTUs of 846 / Holcus lanatus / Flowers
OTUs of 468 / Holcus lanatus / Leaves
OTUs of 471 / Holcus lanatus / Leaves
OTUs of 489 / Holcus lanatus / Leaves
OTUs of 492 / Holcus lanatus / Leaves
OTUs of 495 / Holcus lanatus / Leaves
OTUs of 496 / Holcus lanatus / Leaves
OTUs of 499 / Holcus lanatus / Leaves
OTUs of 504 / Holcus lanatus / Leaves
OTUs of 508 / Holcus lanatus / Leaves
OTUs of 509 / Holcus lanatus / Leaves
OTUs of 534 / Holcus lanatus / Leaves
OTUs of 537 / Holcus lanatus / Leaves
OTUs of 540 / Holcus lanatus / Leaves
OTUs of 559 / Holcus lanatus / Leaves
OTUs of 562 / Holcus lanatus / Leaves
OTUs of 567 / Holcus lanatus / Leaves
OTUs of 583 / Holcus lanatus / Leaves
OTUs of 586 / Holcus lanatus / Leaves
OTUs of 588 / Holcus lanatus / Leaves
OTUs of 592 / Holcus lanatus / Leaves
OTUs of 595 / Holcus lanatus / Leaves
OTUs of 598 / Holcus lanatus / Leaves
OTUs of 601 / Holcus lanatus / Leaves
OTUs of 604 / Holcus lanatus / Leaves
OTUs of 653 / Holcus lanatus / Leaves
OTUs of 656 / Holcus lanatus / Leaves
OTUs of 659 / Holcus lanatus / Leaves
OTUs of 662 / Holcus lanatus / Leaves
OTUs of 667 / Holcus lanatus / Leaves
OTUs of 668 / Holcus lanatus / Leaves
OTUs of 671 / Holcus lanatus / Leaves
OTUs of 674 / Holcus lanatus / Leaves
OTUs of 698 / Holcus lanatus / Leaves
OTUs of 701 / Holcus lanatus / Leaves
OTUs of 704 / Holcus lanatus / Leaves
