# Supplementary material for: Consistent host and organ occupancy of phyllosphere bacteria in a community of wild herbaceous plant species
Source: ISME J. 2019 Oct 17;14(1):245–58. doi: 10.1038/s41396-019-0531-8 (PMC6908658; doi:10.1038/s41396-019-0531-8)
Supplement: Supplementary file 14 — Supplementary figure 6 [file 41396_2019_531_MOESM14_ESM.pdf]

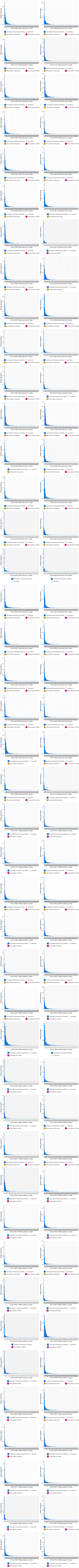
Relative abundance
As prevalent on Ranunculus and Trifolium
Inconclusive
More prevalent on Ranunculus
More prevalent on Trifolium
OTUs of 466 / Ranunculus acris / Leaves
OTUs of 469 / Ranunculus acris / Leaves
OTUs of 472 / Ranunculus acris / Leaves
OTUs of 475 / Ranunculus acris / Leaves
OTUs of 488 / Ranunculus acris / Leaves
OTUs of 491 / Ranunculus acris / Leaves
OTUs of 494 / Ranunculus acris / Leaves
OTUs of 497 / Ranunculus acris / Leaves
OTUs of 501 / Ranunculus acris / Leaves
OTUs of 503 / Ranunculus acris / Leaves
OTUs of 505 / Ranunculus acris / Leaves
OTUs of 507 / Ranunculus acris / Leaves
OTUs of 529 / Ranunculus acris / Leaves
OTUs of 532 / Ranunculus acris / Leaves
OTUs of 535 / Ranunculus acris / Leaves
OTUs of 538 / Ranunculus acris / Leaves
OTUs of 557 / Ranunculus acris / Leaves
OTUs of 560 / Ranunculus acris / Leaves
OTUs of 563 / Ranunculus acris / Leaves
OTUs of 566 / Ranunculus acris / Leaves
OTUs of 581 / Ranunculus acris / Leaves
OTUs of 584 / Ranunculus acris / Leaves
OTUs of 589 / Ranunculus acris / Leaves
OTUs of 590 / Ranunculus acris / Leaves
OTUs of 593 / Ranunculus acris / Leaves
OTUs of 596 / Ranunculus acris / Leaves
OTUs of 599 / Ranunculus acris / Leaves
OTUs of 602 / Ranunculus acris / Leaves
OTUs of 629 / Ranunculus acris / Leaves
OTUs of 632 / Ranunculus acris / Leaves
OTUs of 635 / Ranunculus acris / Leaves
OTUs of 639 / Ranunculus acris / Leaves
OTUs of 655 / Ranunculus acris / Leaves
OTUs of 657 / Ranunculus acris / Leaves
OTUs of 661 / Ranunculus acris / Leaves
OTUs of 663 / Ranunculus acris / Leaves
OTUs of 665 / Ranunculus acris / Leaves
OTUs of 669 / Ranunculus acris / Leaves
OTUs of 675 / Ranunculus acris / Leaves
OTUs of 676 / Ranunculus acris / Leaves
OTUs of 693 / Ranunculus acris / Leaves
OTUs of 696 / Ranunculus acris / Leaves
OTUs of 699 / Ranunculus acris / Leaves
OTUs of 702 / Ranunculus acris / Leaves
OTUs of 467 / Trifolium pratense / Leaves
OTUs of 470 / Trifolium pratense / Leaves
OTUs of 473 / Trifolium pratense / Leaves
OTUs of 487 / Trifolium pratense / Leaves
OTUs of 490 / Trifolium pratense / Leaves
OTUs of 493 / Trifolium pratense / Leaves
OTUs of 498 / Trifolium pratense / Leaves
OTUs of 500 / Trifolium pratense / Leaves
OTUs of 502 / Trifolium pratense / Leaves
OTUs of 506 / Trifolium pratense / Leaves
OTUs of 510 / Trifolium pratense / Leaves
OTUs of 530 / Trifolium pratense / Leaves
OTUs of 533 / Trifolium pratense / Leaves
OTUs of 536 / Trifolium pratense / Leaves
OTUs of 539 / Trifolium pratense / Leaves
OTUs of 558 / Trifolium pratense / Leaves
OTUs of 561 / Trifolium pratense / Leaves
OTUs of 564 / Trifolium pratense / Leaves
OTUs of 568.2 / Trifolium pratense / Leaves
OTUs of 582 / Trifolium pratense / Leaves
OTUs of 585 / Trifolium pratense / Leaves
OTUs of 587 / Trifolium pratense / Leaves
OTUs of 591.2 / Trifolium pratense / Leaves
OTUs of 594 / Trifolium pratense / Leaves
OTUs of 597 / Trifolium pratense / Leaves
OTUs of 600 / Trifolium pratense / Leaves
OTUs of 603 / Trifolium pratense / Leaves
OTUs of 630 / Trifolium pratense / Leaves
OTUs of 633 / Trifolium pratense / Leaves
OTUs of 636 / Trifolium pratense / Leaves
OTUs of 640 / Trifolium pratense / Leaves
OTUs of 658 / Trifolium pratense / Leaves
OTUs of 660 / Trifolium pratense / Leaves
OTUs of 664 / Trifolium pratense / Leaves
OTUs of 666 / Trifolium pratense / Leaves
OTUs of 670 / Trifolium pratense / Leaves
OTUs of 672 / Trifolium pratense / Leaves
OTUs of 673 / Trifolium pratense / Leaves
OTUs of 694 / Trifolium pratense / Leaves
OTUs of 697 / Trifolium pratense / Leaves
OTUs of 700 / Trifolium pratense / Leaves
OTUs of 703 / Trifolium pratense / Leaves
