# Supplementary material for: Consistent host and organ occupancy of phyllosphere bacteria in a community of wild herbaceous plant species
Source: ISME J. 2019 Oct 17;14(1):245–58. doi: 10.1038/s41396-019-0531-8 (PMC6908658; doi:10.1038/s41396-019-0531-8)
Supplement: Supplementary file 15 — Supplementary figure 7 [file 41396_2019_531_MOESM15_ESM.pdf]

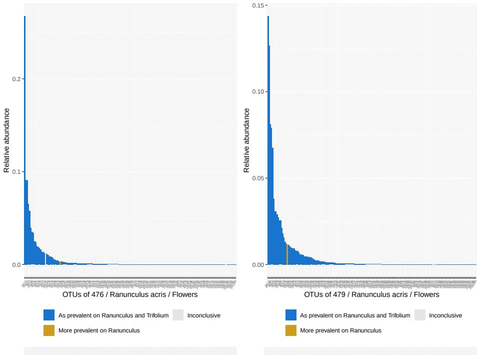
0.2
0.1
0.0
Relative abundance
OTUs of 476 / Ranunculus acris / Flowers
As prevalent on Ranunculus and Trifolium
Inconclusive
More prevalent on Ranunculus
0.15
0.10
0.05
0.00
Relative abundance
OTUs of 479 / Ranunculus acris / Flowers
As prevalent on Ranunculus and Trifolium
Inconclusive
More prevalent on Ranunculus

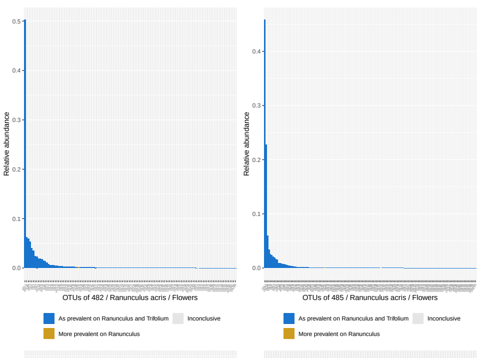
0.5
0.4
0.3
0.2
0.1
0.0
Relative abundance
OTUs of 482 / Ranunculus acris / Flowers
As prevalent on Ranunculus and Trifolium
Inconclusive
More prevalent on Ranunculus
0.4
0.3
0.2
0.1
0.0
Relative abundance
OTUs of 485 / Ranunculus acris / Flowers
As prevalent on Ranunculus and Trifolium
Inconclusive
More prevalent on Ranunculus

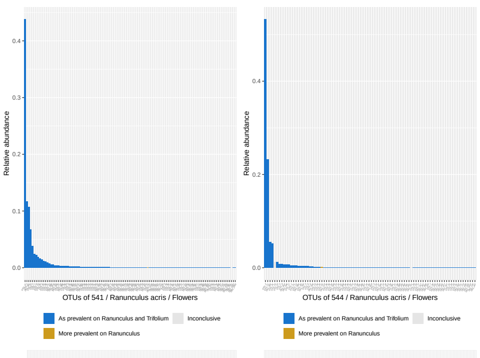
0.4
0.3
0.2
0.1
0.0
Relative abundance
OTUs of 541 / Ranunculus acris / Flowers
As prevalent on Ranunculus and Trifolium
Inconclusive
More prevalent on Ranunculus
0.4
0.2
0.0
Relative abundance
OTUs of 544 / Ranunculus acris / Flowers
As prevalent on Ranunculus and Trifolium
Inconclusive
More prevalent on Ranunculus

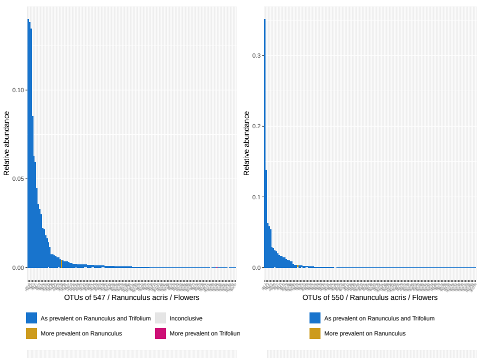
0.10
0.05
0.00
Relative abundance
OTUs of 547 / Ranunculus acris / Flowers
As prevalent on Ranunculus and Trifolium
Inconclusive
More prevalent on Ranunculus
More prevalent on Trifolium
0.3
0.2
0.1
0.0
Relative abundance
OTUs of 550 / Ranunculus acris / Flowers
As prevalent on Ranunculus and Trifolium
More prevalent on Ranunculus

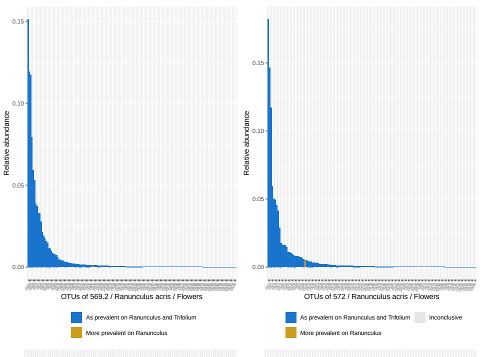
0.15
0.10
0.05
0.00
Relative abundance
OTUs of 569.2 / Ranunculus acris / Flowers
As prevalent on Ranunculus and Trifolium
More prevalent on Ranunculus
0.15
0.10
0.05
0.00
Relative abundance
OTUs of 572 / Ranunculus acris / Flowers
As prevalent on Ranunculus and Trifolium
Inconclusive
More prevalent on Ranunculus

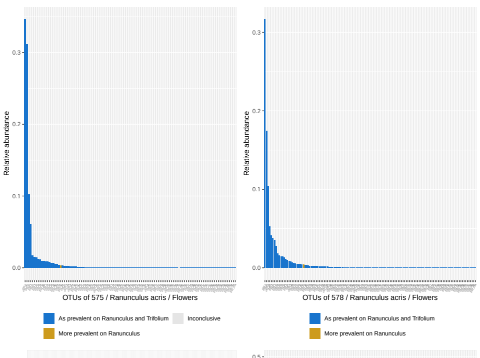
0.3
0.2
0.1
0.0
Relative abundance
OTUs of 575 / Ranunculus acris / Flowers
As prevalent on Ranunculus and Trifolium
Inconclusive
More prevalent on Ranunculus
0.3
0.2
0.1
0.0
Relative abundance
OTUs of 578 / Ranunculus acris / Flowers
As prevalent on Ranunculus and Trifolium
More prevalent on Ranunculus

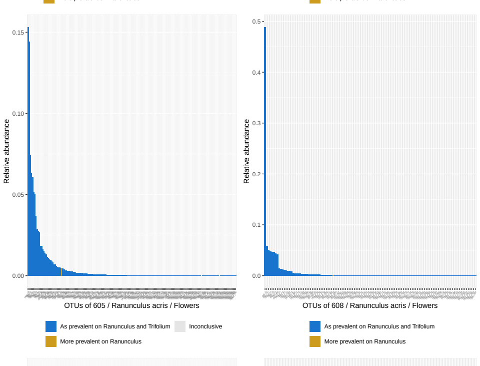
0.15
0.10
0.05
0.00
Relative abundance
OTUs of 605 / Ranunculus acris / Flowers
As prevalent on Ranunculus and Trifolium
Inconclusive
More prevalent on Ranunculus
0.5
0.4
0.3
0.2
0.1
0.0
Relative abundance
OTUs of 608 / Ranunculus acris / Flowers
As prevalent on Ranunculus and Trifolium
More prevalent on Ranunculus

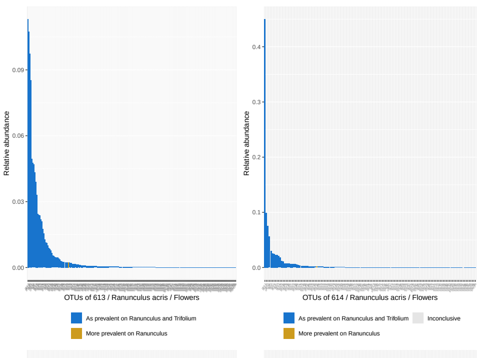
0.09
0.06
0.03
0.00
Relative abundance
OTUs of 613 / Ranunculus acris / Flowers
As prevalent on Ranunculus and Trifolium
More prevalent on Ranunculus
0.4
0.3
0.2
0.1
0.0
Relative abundance
OTUs of 614 / Ranunculus acris / Flowers
As prevalent on Ranunculus and Trifolium
Inconclusive
More prevalent on Ranunculus

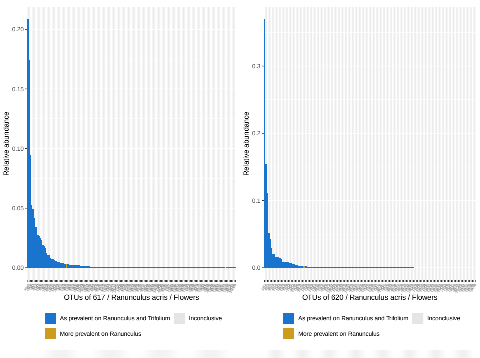
0.20
0.15
0.10
0.05
0.00
Relative abundance
OTUs of 617 / Ranunculus acris / Flowers
As prevalent on Ranunculus and Trifolium
Inconclusive
More prevalent on Ranunculus
0.3
0.2
0.1
0.0
Relative abundance
OTUs of 620 / Ranunculus acris / Flowers
As prevalent on Ranunculus and Trifolium
Inconclusive
More prevalent on Ranunculus

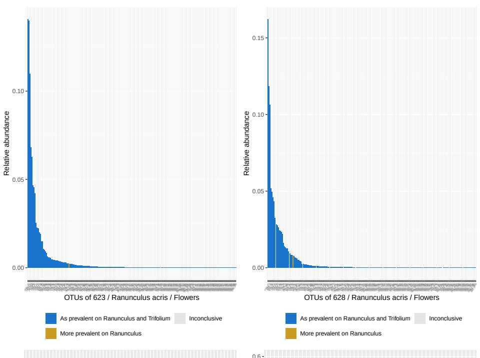
0.10
0.05
0.00
Relative abundance
OTUs of 623 / Ranunculus acris / Flowers
As prevalent on Ranunculus and Trifolium
Inconclusive
More prevalent on Ranunculus
0.15
0.10
0.05
0.00
Relative abundance
OTUs of 628 / Ranunculus acris / Flowers
As prevalent on Ranunculus and Trifolium
Inconclusive
More prevalent on Ranunculus

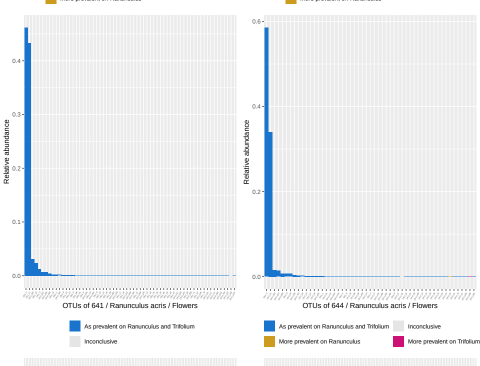
0.4
0.3
0.2
0.1
0.0
Relative abundance
OTUs of 641 / Ranunculus acris / Flowers
As prevalent on Ranunculus and Trifolium
Inconclusive
0.6
0.4
0.2
0.0
Relative abundance
OTUs of 644 / Ranunculus acris / Flowers
As prevalent on Ranunculus and Trifolium
Inconclusive
More prevalent on Ranunculus
More prevalent on Trifolium

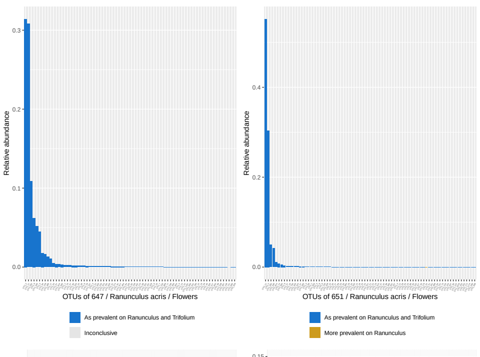
0.3
0.2
0.1
0.0
Relative abundance
OTUs of 647 / Ranunculus acris / Flowers
As prevalent on Ranunculus and Trifolium
Inconclusive
0.4
0.2
0.0
Relative abundance
OTUs of 651 / Ranunculus acris / Flowers
As prevalent on Ranunculus and Trifolium
More prevalent on Ranunculus

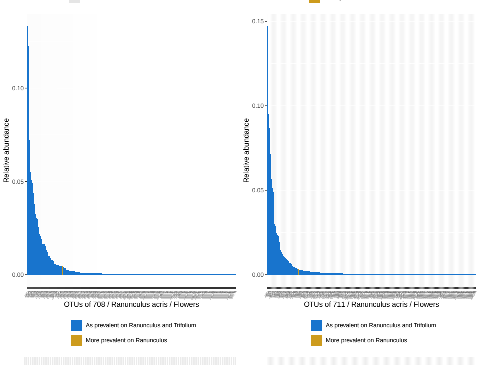
0.10
0.05
0.00
Relative abundance
OTUs of 708 / Ranunculus acris / Flowers
As prevalent on Ranunculus and Trifolium
More prevalent on Ranunculus
0.15
0.10
0.05
0.00
Relative abundance
OTUs of 711 / Ranunculus acris / Flowers
As prevalent on Ranunculus and Trifolium
More prevalent on Ranunculus

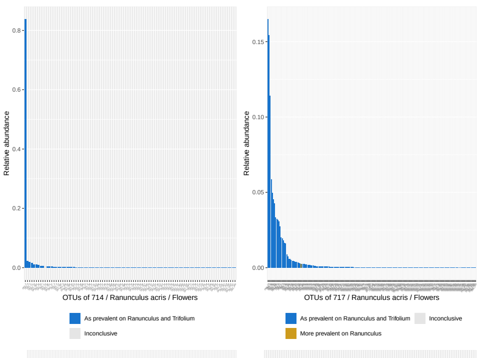
0.8
0.6
0.4
0.2
0.0
Relative abundance
OTUs of 714 / Ranunculus acris / Flowers
As prevalent on Ranunculus and Trifolium
Inconclusive
0.15
0.10
0.05
0.00
Relative abundance
OTUs of 717 / Ranunculus acris / Flowers
As prevalent on Ranunculus and Trifolium
Inconclusive
More prevalent on Ranunculus

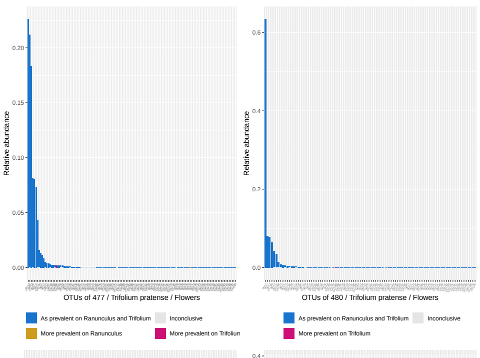
0.20
0.15
0.10
0.05
0.00
Relative abundance
OTUs of 477 / Trifolium pratense / Flowers
As prevalent on Ranunculus and Trifolium
Inconclusive
More prevalent on Ranunculus
More prevalent on Trifolium
0.6
0.4
0.2
0.0
Relative abundance
OTUs of 480 / Trifolium pratense / Flowers
As prevalent on Ranunculus and Trifolium
Inconclusive
More prevalent on Trifolium

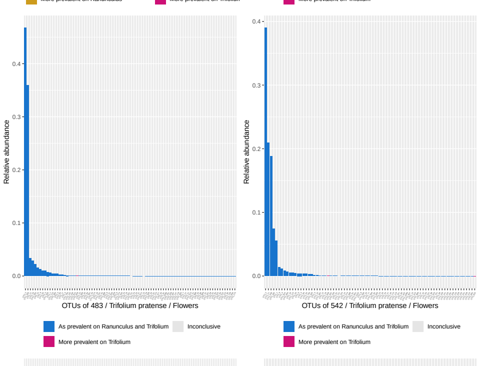
0.4
0.3
0.2
0.1
0.0
Relative abundance
OTUs of 483 / Trifolium pratense / Flowers
As prevalent on Ranunculus and Trifolium
Inconclusive
More prevalent on Trifolium
0.4
0.3
0.2
0.1
0.0
Relative abundance
OTUs of 542 / Trifolium pratense / Flowers
As prevalent on Ranunculus and Trifolium
Inconclusive
More prevalent on Trifolium

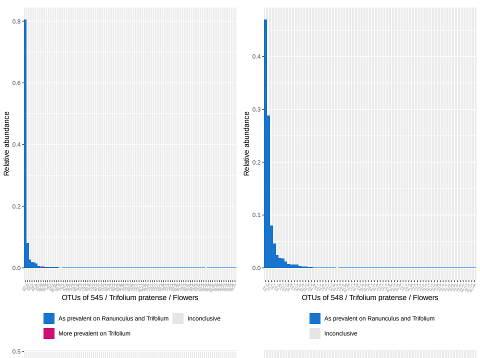
0.8
0.6
0.4
0.2
0.0
Relative abundance
OTUs of 545 / Trifolium pratense / Flowers
As prevalent on Ranunculus and Trifolium
Inconclusive
More prevalent on Trifolium
0.4
0.3
0.2
0.1
0.0
Relative abundance
OTUs of 548 / Trifolium pratense / Flowers
As prevalent on Ranunculus and Trifolium
Inconclusive

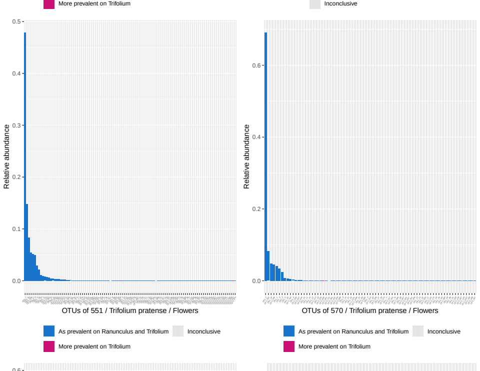
0.5
0.4
0.3
0.2
0.1
0.0
Relative abundance
OTUs of 551 / Trifolium pratense / Flowers
As prevalent on Ranunculus and Trifolium
Inconclusive
More prevalent on Trifolium
0.6
0.4
0.2
0.0
Relative abundance
OTUs of 570 / Trifolium pratense / Flowers
As prevalent on Ranunculus and Trifolium
Inconclusive
More prevalent on Trifolium

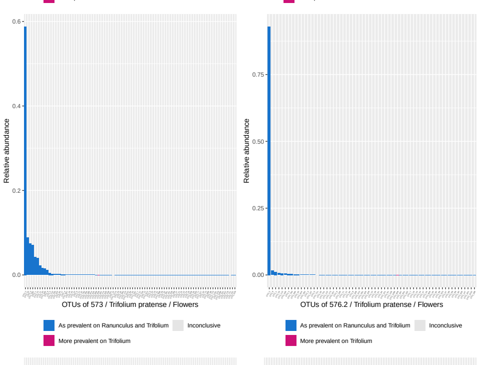
0.6
0.4
0.2
0.0
Relative abundance
OTUs of 573 / Trifolium pratense / Flowers
As prevalent on Ranunculus and Trifolium
Inconclusive
More prevalent on Trifolium
0.75
0.50
0.25
0.00
Relative abundance
OTUs of 576.2 / Trifolium pratense / Flowers
As prevalent on Ranunculus and Trifolium
Inconclusive
More prevalent on Trifolium

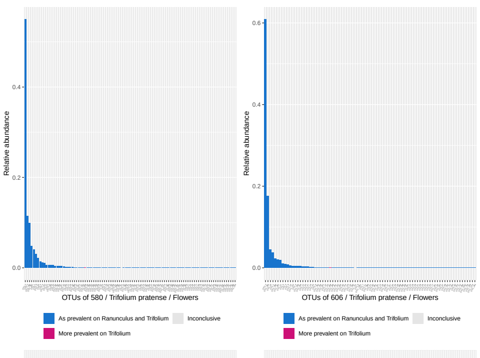
0.4
0.2
0.0
Relative abundance
OTUs of 580 / Trifolium pratense / Flowers
As prevalent on Ranunculus and Trifolium
Inconclusive
More prevalent on Trifolium
0.6
0.4
0.2
0.0
Relative abundance
OTUs of 606 / Trifolium pratense / Flowers
As prevalent on Ranunculus and Trifolium
Inconclusive
More prevalent on Trifolium

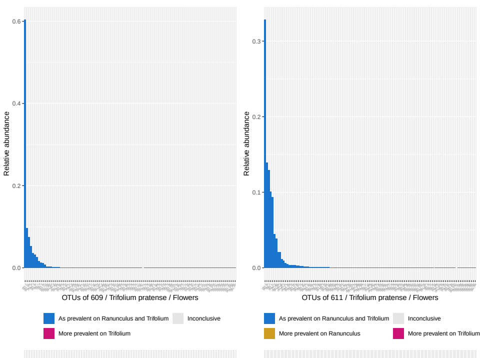
0.6
0.4
0.2
0.0
Relative abundance
OTUs of 609 / Trifolium pratense / Flowers
As prevalent on Ranunculus and Trifolium
Inconclusive
More prevalent on Trifolium
0.3
0.2
0.1
0.0
Relative abundance
OTUs of 611 / Trifolium pratense / Flowers
As prevalent on Ranunculus and Trifolium
Inconclusive
More prevalent on Ranunculus
More prevalent on Trifolium

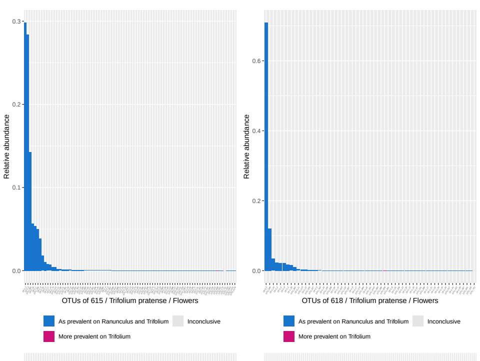
0.3
0.2
0.1
0.0
Relative abundance
OTUs of 615 / Trifolium pratense / Flowers
As prevalent on Ranunculus and Trifolium
Inconclusive
More prevalent on Trifolium
0.6
0.4
0.2
0.0
Relative abundance
OTUs of 618 / Trifolium pratense / Flowers
As prevalent on Ranunculus and Trifolium
Inconclusive
More prevalent on Trifolium

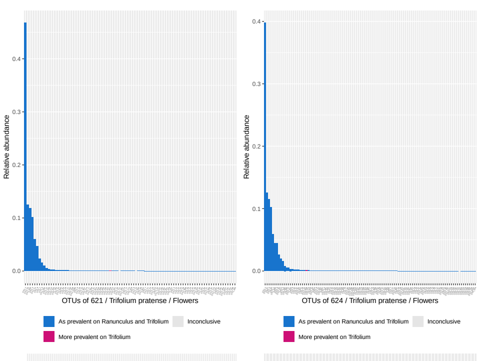
0.4
0.3
0.2
0.1
0.0
Relative abundance
OTUs of 621 / Trifolium pratense / Flowers
As prevalent on Ranunculus and Trifolium
Inconclusive
More prevalent on Trifolium
0.4
0.3
0.2
0.1
0.0
Relative abundance
OTUs of 624 / Trifolium pratense / Flowers
As prevalent on Ranunculus and Trifolium
Inconclusive
More prevalent on Trifolium

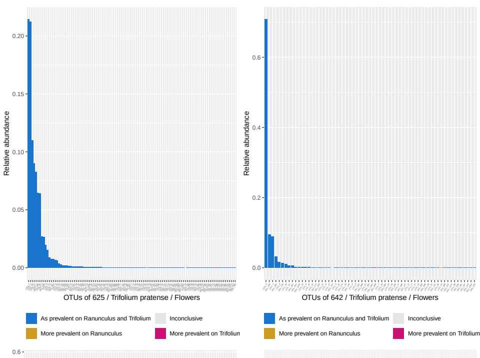
0.20
0.15
0.10
0.05
0.00
Relative abundance
OTUs of 625 / Trifolium pratense / Flowers
As prevalent on Ranunculus and Trifolium
Inconclusive
More prevalent on Ranunculus
More prevalent on Trifolium
0.6
0.4
0.2
0.0
Relative abundance
OTUs of 642 / Trifolium pratense / Flowers
As prevalent on Ranunculus and Trifolium
Inconclusive
More prevalent on Ranunculus
More prevalent on Trifolium

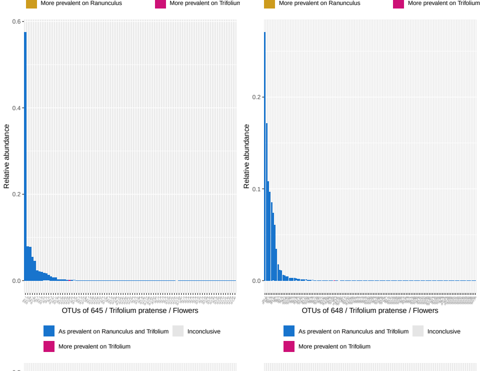
0.6
0.4
0.2
0.0
Relative abundance
OTUs of 645 / Trifolium pratense / Flowers
As prevalent on Ranunculus and Trifolium
Inconclusive
More prevalent on Trifolium
0.2
0.1
0.0
Relative abundance
OTUs of 648 / Trifolium pratense / Flowers
As prevalent on Ranunculus and Trifolium
Inconclusive
More prevalent on Trifolium

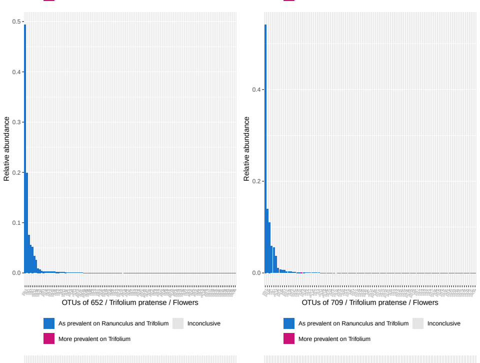
0.5
0.4
0.3
0.2
0.1
0.0
Relative abundance
OTUs of 652 / Trifolium pratense / Flowers
As prevalent on Ranunculus and Trifolium
Inconclusive
More prevalent on Trifolium
0.4
0.2
0.0
Relative abundance
OTUs of 709 / Trifolium pratense / Flowers
As prevalent on Ranunculus and Trifolium
Inconclusive
More prevalent on Trifolium

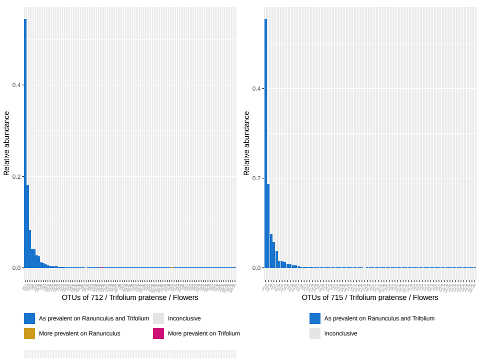
0.4
0.2
0.0
Relative abundance
OTUs of 712 / Trifolium pratense / Flowers
As prevalent on Ranunculus and Trifolium
Inconclusive
More prevalent on Ranunculus
More prevalent on Trifolium
0.4
0.2
0.0
Relative abundance
OTUs of 715 / Trifolium pratense / Flowers
As prevalent on Ranunculus and Trifolium
Inconclusive

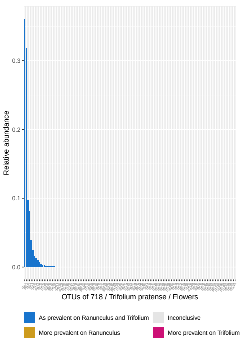
0.3
0.2
0.1
0.0
Relative abundance
OTUs of 718 / Trifolium pratense / Flowers
As prevalent on Ranunculus and Trifolium
Inconclusive
More prevalent on Ranunculus
More prevalent on Trifolium
